# HpaR, the Repressor of Aromatic Compound Metabolism, Positively Regulates the Expression of T6SS4 to Resist Oxidative Stress in *Yersinia pseudotuberculosis*

**DOI:** 10.3389/fmicb.2020.00705

**Published:** 2020-04-17

**Authors:** Zhuo Wang, Tietao Wang, Rui Cui, Zhenxing Zhang, Keqi Chen, Mengyun Li, Yueyue Hua, Huawei Gu, Lei Xu, Yao Wang, Yantao Yang, Xihui Shen

**Affiliations:** State Key Laboratory of Crop Stress Biology for Arid Areas, Shaanxi Key Laboratory of Agricultural and Environmental Microbiology, College of Life Sciences, Northwest A&F University, Yangling, China

**Keywords:** HpaR, type VI secretion system, *Yersinia pseudotuberculosis*, oxidative stress, biofilm, aromatic compounds degradation

## Abstract

HpaR, a MarR family transcriptional regulator, was first identified in *Escherichia coli* W for its regulation of the *hpa-meta* operon. Little else is known regarding its functionality. Here, we report that in *Yersinia pseudotuberculosis*, HpaR negatively regulates the *hpa-meta* operon similar to in *E. coli* W. To investigate additional functions of HpaR, RNA sequencing was performed for both the wild-type and the Δ*hpaR* mutant, which revealed that the type VI secretion system (T6SS) was positively regulated by HpaR. T6SS4 is important for bacteria resisting environmental stress, especially oxidative stress. We demonstrate that HpaR facilitates bacteria resist oxidative stress by upregulating the expression of T6SS4 in *Y*. *pseudotuberculosis*. HpaR is also involved in biofilm formation, antibiotic resistance, adhesion to eukaryotic cells, and virulence in mice. These results greatly expand our knowledge of the functionality of HpaR and reveal a new pathway that regulates T6SS4.

## Introduction

HpaR has been characterized as a repressor of the *hpa-meta* operon (Galan et al., 2003). It has been shown that *Escherichia coli* strains B, C, and W can use 4-hydroxyphenylacetic acid (4-HPA) as a carbon source via the *hpa-meta* pathway, while *E*. *coli* K-12 does not have this ability (Cooper and Skinner, 1980). Studies of the *hpa-meta* cluster have focused mainly on *E*. *coli* W (Galan et al., 2001). The *hpa-meta* cluster of *E*. *coli* W is composed of 11 genes in two putative operons (Prieto et al., 1996; Galan et al., 2003). The *upper* operon consists of *hpaBC*, which encodes the two-component 4-HPA monooxygenase, which converts 4-HPA to 3,4-dihydroxyphenylacetic acid (3,4-HPA) (Prieto and Garcia, 1994; Galan et al., 2000). Transcription of the *hpaBC* operon is controlled by HpaA, which belongs to the AraC/XylS family and functions as an activator to activate the expression of *hpaBC*, except for the *meta* operon in the presence of 4-HPA or 3-HPA (Prieto and Garcia, 1997). The *meta* operon is composed of *hpaGEDFHI*, which encodes enzymes that catalyze 3,4-HPA in the Krebs cycle (Roper et al., 1993; Prieto et al., 1996). The expression of both the *meta* operon and *hpaR* are regulated by HpaR (Galan et al., 2003). HpaR was first identified as a repressor in *E*. *coli* W and is identical to HpcR in *E*. *coli* C (Roper et al., 1993; Galan et al., 2003). The *hpa-meta* cluster has also been found in other bacteria. In *Pseudomonas putida* U, the *hpa* pathway is coupled with the *tyn* pathway to degrade tyramine and dopamine (Barbour and Bayly, 1981; Arcos et al., 2010). Together these form the 3,4-HPA catabolon, in which 3,4-HPA is the central intermediate. The *hpa-meta* pathway in *P*. *putida* U is composed of the genes *hpaRBCIHXFDEG2G1AY*. HpaR of *P*. *putida* U is similar to that in *E*. *coli* in that it represses the *meta* operon (Arcos et al., 2010). Interestingly, a second *hpaR* (*hpaY*) was found in *P*. *putida* U with similar functionality, ensuring stronger control of the *meta* operon (Arcos et al., 2010). In *Burkholderia xenovorans* LB400, the *hpa-meta* pathway is made up of *hpaG1G2EDFHI* and *hpaBC*, while *hpaA* and *hpaX* are absent and *hpaBC* is not adjacent to the *meta* operon (Mendez et al., 2011). HpaR of *B*. *xenovorans* LB400 also regulates the *meta* operon as a repressor (Mendez et al., 2011). However, previous research has defined HpaR as a repressor that negatively controls expression of the *meta* operon, but not *hpaBC*. According to sequence comparison and a three-dimensional model, HpaR is classified into the MarR transcriptional regulator family, which is involved in various physiological processes (Alekshun et al., 2001; Galan et al., 2003; Grove, 2013). However, whether HpaR might exert other functions in addition to acting as a repressor of the *hpa-meta* operon remains unknown.

*Yersinia pseudotuberculosis* is a Gram-negative enteric pathogen of animals and humans that causes a variety of diseases such as acute ileitis, mesenteric lymphadenitis, and septicemia (Brubaker, 1991; Smego et al., 1999; Fallman and Gustavsson, 2005). During infection, environmental stress and host immunity reactions can cause an increase in reactive oxygen species (ROS) levels of *Y*. *pseudotuberculosis* (Green et al., 2016). Elevated cellular ROS levels lead to oxidative stress, which induces oxidative damage to macromolecules such as proteins, lipids, and DNA (D’Autreaux and Toledano, 2007). Protection against the adverse effects of ROS is vital—bacteria have developed a wide range of systems including antioxidant enzymes, i.e., peroxidase, superoxide dismutase, glutaredoxin, and thioredoxin; low molecular weight antioxidants, i.e., the tripeptide glutathione (GSH) and β-carotene; and vitamins, i.e., vitamins C and E (D’Autreaux and Toledano, 2007; Si et al., 2015, 2017a; Staerck et al., 2017). Recently, we found that the type VI secretion system (T6SS) in *Y. pseudotuberculosis* was also involved in resistance to oxidative stress; it secretes a zinc-binding protein that imports zinc to mitigate ROS (Wang et al., 2015).

T6SS is a versatile transmembrane machine used by many Gram-negative bacteria to inject effector proteins into cells, either prokaryotic or eukaryotic, or the extracellular milieu (Durand et al., 2014; Russell et al., 2014; Basler, 2015). Although traditionally T6SS is recognized as a contact-dependent bacterial weapon for interspecies competition, some T6SSs from diverse species are also found to play roles in bacterial pathogenesis, biofilm formation, and stress response (Durand et al., 2014; Russell et al., 2014; Yang et al., 2018). For example, a *Vibrio anguillarum* T6SS regulated by the general stress response regulator RpoS is involved in resistance to hydrogen peroxide, ethanol, and low pH (Weber et al., 2009). Enterohemorrhagic *E*. *coli* (EHEC) uses its T6SS to deliver KatN, an Mn-containing catalase, into the host cytosol, resulting in reduced levels of intracellular ROS and greater survival of the pathogen (Wan et al., 2017). In *B*. *thailandensis*, T6SS4 exports TseZ and TseM into the extracellular medium to acquire the antioxidant metal ions Zn^2+^ and Mn^2+^, respectively, to combat oxidative stress by reducing intracellular ROS levels (Si et al., 2017b,c). The avian pathogenic *E*. *coli* (APEC) strain TW-XM harbors two functional T6SSs. The first, T6SS1, plays versatile roles in adherence to host cells, biofilm formation, and bacterial competition; the second, T6SS2, is responsible only for cerebral infection (Ma et al., 2014; Navarro-Garcia et al., 2019).

Despite being reported to control expression of the *hpa-meta* operon, knowledge of HpaR in *Y*. *pseudotuberculosis* is lacking. Unexpectedly, in this study, we found that HpaR not only acts as a repressor of the *hpa-meta* operon in a similar manner to that of *E*. *coli* but also acts as an activator to upregulate the expression of T6SS4 to acquire Zn^2+^ and resist oxidative stress. HpaR is also involved in biofilm formation, antibiotic resistance, adhesion to eukaryotic cells, and virulence in mice. These results greatly expand our current knowledge of the functions of HpaR.

## Materials and Methods

### Bacterial Strains and Growth Conditions

Bacterial strains and plasmids used in this study are listed in Supplementary Table S1. *Escherichia coli* strains were cultured at 37°C in Lysogeny Broth (LB) or LB plates. *Y. pseudotuberculosis* strains were grown in Yersinia-Lysogeny-Broth (YLB) broth (tryptone 1%, yeast extract 0.5%, NaCl 0.5%) or YLB plates at 30 or 26°C. The *Y. pseudotuberculosis strain* YPIII was the parent of all derivatives used in this study. To construct the *hpaR* in-frame deletion mutant, the wild-type *Y. pseudotuberculosis* was mated with *E. coli* S17-1λpir carrying pDM4-*hpaR* and chromosomal integration was selected by plating on YLB agar plates supplemented with nalidixic acid and chloramphenicol. The *hpaR* deletion mutant was subsequently screened on YLB agar plates with 20% sucrose and confirmed by polymerase chain reaction (PCR) and DNA sequencing. Appropriate antibiotics were included in growth medium at the following concentrations: ampicillin, 100 μg/ml; kanamycin, 50 μg/ml; nalidixic acid, 20 μg/ml; chloramphenicol, 20 μg/ml.

### Plasmid Construction

Primers used in this study are listed in Supplementary Table S2. The *lacZ* fusion reporter vector pDM4-T6SS4*p:lacZ* was made in previous study (Zhang et al., 2013). To obtain the expression plasmid, *hpaR*-F-*Bam*HI/*hpaR*-R-*Sal*I primer pair was used to amplify *hpaR* gene fragment from *Y. pseudotuberculosis* genomic DNA by PCR. The *hpaR* gene fragment were digested with *Bam*HI/*Sal*I and then inserted into similar digested pET28a yielding the pET28a-*hpaR*. The suicide plasmid pDM4-*hpaR* used to construct the *hpaR* mutant was prepared by overlap PCR. Briefly, *hpaR*-UF-*Xba*I/*hpaR*-UR and *hpaR*-DF/*hpaR*-DR-*Spe*I primers were used to amplify the 1000 bp upstream fragment and 1000 bp downstream fragment of *hpaR*, respectively. Then, the amplified fragments were fused together by overlap PCR with *hpaR*-UF-*Xba*I/*hpaR*-DR-*Spe*I. The fused PCR products were digested with *Xba*I/*Spe*I and cloned into similar digested pDM4 resulting pDM4-*hpaR*. In order to construct the complementary plasmid pKT100-*hpaR*, *hpaR*-F-*Bam*HI/*hpaR*-R-*Sal*I primers were used to amplify *hpaR* gene from template. The PCR product was digested with *Bam*HI/*Sal*I and inserted into similarly digested pKT100 to produce pKT100-*hpaR*, which is subsequently electroporated into Δ*hpaR* to construct the complementation strains. To construct the *hpaG1* promoter reporter vector, *hpaG1p*-F-*Sal*I/*hpaG1p*-R-*Xba*I primer pair was used to amplify the 387 bp promoter fragment from *Y. pseudotuberculosis*. The product of PCR was digested with *Sal*I/*Xba*I and cloned into similar digested pDM4-T6SS4*p:lacZ* to produce pDM4-*hpaG1p:lacZ*. To construct the T6SS4 promoter fragments with HpaR binding site mutations, overlap PCR was performed to replace the HpaR binding site with identical amount of irrelevant base pairs. T6SS4*p*M_*HpaR*_-R and T6SS4*p*M_*HpaR*_-F were designed to contain 22 bp overlapping DNA fragment (ATTTGTTAGATTCCGAACCGTC) used to replace the HpaR binding site. T6SS4*p*-F-*Sal*I/T6SS4*p*M_*HpaR*_-R and T6SS4*p*M_*HpaR*_-F/T6SS4*p*-R-*Xba*I primers used to amplify the up-fragment and down-fragment of T6SS4*p* promoter, respectively. The PCR products were ligated with T6SS4*p*-F-*Sal*I/T6SS4*p*-R-*Xba*I by overlap PCR to produce the mutant promoter fragment T6SS4*p*M_*HpaR*_. The T6SS4*p*M_*HpaR*_ fragment were digested with *Sal*I/*Xba*I and ligated into similarly digested pDM4-T6SS4*p:lacZ* to produce pDM4-T6SS4*p*M_*HpaR*_*:lacZ*. The validity of all the plasmids constructed above was confirmed by DNA sequencing.

### Sequence Data Analysis

Sequence alignment was performed as previously described (Shen et al., 2005; Huang et al., 2008). The sequence of *hpaRGEDFHIXABC* (Accession No. Z37980.2) of *E. coli* W were retrieved from GenBank. Sequence comparisons were carried out using BLAST program at National Center for Biotechnology Information website^1^. Multiple protein sequence alignment was made by CLUSTAL W. The sequences used in alignment have been deposited in the GenBank database [Accession No. EcC *E. coli* C (S56952.1), EcW *E. coli* W (Z37980.2), Yptb *Y. pseudotuberculosis* YPIII (ACA68739.1), PpU *P. putida* U (FJ904934.1), BxLB400 *B. xenovorans* LB400 (ABE33958.1)]. The result was exported by ESPript^2^.

### Construction of Chromosomal Fusion Reporter Strains and β-Galactosidase Activity Assay

The *lacZ* fusion reporter vector pDM4-T6SS4*p:lacZ*, pDM4-T6SS4*p*M_*HpaR*_*:lacZ* and pDM4-*hpaG1p:lacZ* was transformed into *E. coli* S17-1λpir and then introduced into *Y. pseudotuberculosis* by conjugation as described (Xu et al., 2014). All constructed *lacZ* fusion reporter strains were grown in YLB broth at 26°C and *o*-nitrophenyl-β-galactoside (ONPG) as substrate for measuring β-galactosidase activities as described by Miller (1992). The β-galactosidase results shown represent the mean of one representative assay performed in triplicate, and error bars represent standard deviation. Statistical analysis was carried out with Student’s *t*-test.

### Protein Expression and Purification

To express and purify His_6_- tagged HpaR, the constructed expression vector pET28a-*hpaR* were transformed into BL21(DE3). Single colony was cultured in 5 ml LB broth at 37°C overnight and diluted 100-fold into 500 ml LB. Until the OD_600_ = 0.5, the culture was shifted to 22°C, induced with 0.3 mM IPTG and further cultivated for 12 h to express the recombinant proteins. Cell pellet was collected by centrifugation, washed and resuspended in His binding buffer, and lysed by sonication. The recombinant proteins were purified with the His⋅Bind Ni-NTA resin (Novagen) according to the manufacturer’s instructions. Eluted recombinant proteins were dialyzed against the appropriate buffer at 4°C for 4 h and then stored at −80°C until used.

### Electrophoretic Mobility Shift Assay (EMSA)

EMSA was performed as previously described (Zhang et al., 2013; Wang et al., 2015). Bio-T6SS4*p*_*HpaR*_-F/Bio-T6SS4*p*_*HpaR*_-R primers were used to amplify the biotin 5′-end-labeled promoter probes (Bio-T6SS4*p*_*HpaR*_) from *Y. pseudotuberculosis* genomic DNA. The unlabeled T6SS4*p*_*HpaR*_ was amplified with T6SS4*p*_*HpaR*_-F/T6SS4*p*_*HpaR*_-R from template, which used as a competitor. In addition, the unrelated protein BSA was used as negative control. All promoter probes were purified by EasyPure Quick Gel Extraction Kit (TransGen Biotech). According to the manufacturer’s protocol (LightShift Chemiluminescent EMSA Kit; Thermo Fisher Scientific), each 20 μl EMSA reaction solution was prepared as follows: 1 × binding buffer, 50 ng poly (dI-dC), 2.5% glycerol, 0.05% Nonidet P-40, 5 mM MgCl_2_, 3 ng Bio-T6SS4*p*_*HpaR*_ DNA, 1 ng T6SS4*p*_*HpaR*_ DNA as competitor, and different concentration of protein (0, 0.6, 0.8, 0.8 μg). Reaction solutions were incubated for 20 min at room temperature. The samples were loaded onto a 6% polyacrylamide native gel and transferred to a Biodyne B nylon membrane (Thermo Fisher Scientific). The biotin-labeled DNA bands were visualized by chemiluminescent substrate according to the manufacturer’s protocol. *hpaG1p*-F/*hpaG1p*-R primers were used to amplify the *hpaG1p* probes from template. An unrelated DNA (URD) in the similar length or bovine serum albumin (BSA) was included in the binding assay system to serve as negative controls. Increasing concentrations of purified His6-HpaR (0, 0.4, 0.8, 1.2, 1.2 μg) were incubated with 20 ng *hpaG1p* probes in EMSA buffer (20 mM Tris-HCl [pH 7.4], 4 mM MgCl_2_, 100 mM NaCl, 1 mM dithiothreitol, 10% glycerol). After incubation for 20 min at 26°C, the sample was subjected to electrophoresis on a 6% polyacrylamide native gel. Then the DNA probe was detected using SYBR green.

### Stress Survival Assay

*Yersinia pseudotuberculosis* strains grown in YLB broth until mid-exponential are diluted 50-fold into M9 medium (Na_2_HPO_4_, 6 g/L; KH_2_PO_4_, 3 g/L; NaCl, 0.5 g/L; NH_4_Cl, 1 g/L; MgSO_4_, 1 mM; CaCl_2_, 0.1 mM; glucose 0.2%) with or without H_2_O_2_ (1.5 mM), cumene hydroperoxide (CHP, 0.5 mM), ampicillin (0.5 μg/ml), gentamicin (0.2 μg/ml) at 26°C for 1 h at 100 rpm. After treatment, the cultures were diluted 1000-fold and plated onto YLB agar plates with nalidixic acid. Colonies were counted after 24 h at 26°C. The survival percentage of *Y. pseudotuberculosis* is calculated by dividing the number of CFU of stressed cells by the number of CFU of cells without stress. All these assays were performed in triplicate at least three times.

### Fluorescence Dye-Based Intracellular ROS Detection

The intracellular ROS levels were detected by the fluorescent reporter dye 5-(and-6)-chloromethyl-2′,7′-dichlorodihydro fluorescein diacetate, acetylester (CM-H_2_DCFDA, Invitrogen) as previously reported (Si et al., 2017c). Briefly, 1 ml culture were collected after treatment, washed with M9 medium and resuspended in 1 ml of M9 medium containing 10 μM CM-H_2_DCFDA. Samples were incubated at 26°C in the dark for 20 min. Then the cells were pelleted, washed with M9 medium and resuspended in 1 ml of PBS. Then 200 μl samples were transferred to a dark 96-well plate. Fluorescence signals were measured using a SpectraMax M2 Plate Reader (Molecular Devices) with excitation/emission wavelengths of 495/520 nm. The results shown represented the mean of one representative assay performed in triplicate, and error bars represent the standard deviation (SD).

### Protein Secretion Assay

Secretion of YezP (encoded by *ypk_3459*) was detected as previously described (Wang et al., 2015). Briefly, all strains were grown in 180 ml YLB broth at 26°C on a rotary shaker (220 rpm) until OD_600_ = 1.6. A 2 ml culture was centrifuged and the cell pellet was resuspended in 100 μl SDS-loading buffer to serve as the total cell pellet sample. A total of 150 ml culture was centrifuged at low speed (5000 rpm) for 10 min to remove most cell pellets, and the supernatant was further centrifuged at high speed (8000 rpm) for 15 min to eliminate bacteria. To remove the remaining bacteria, the supernatant was filtered through a 0.22-μm filter (Millipore, Billerica, MA, United States). The supernatant was filtered three times through a nitrocellulose filter (BA85) (Whatman) to collect all secretion proteins. The filter was cut into pieces in 1.5 ml tubes and resuspended in 100 μl SDS-loading buffer for 20 min at 65°C to recover the proteins. All samples were normalized to the OD_600_ of the culture and volume used in preparation.

### Western Blot Analysis

Western blot analysis was performed as described previously (Shen et al., 2009; Lin et al., 2017). Samples were resuspended in SDS-Loading buffer and separated in 15% polyacrylamide gel, then transferred onto PVDF membranes (Millipore). The membrane was blocked in 5% (w/v) BSA for 4 h at room temperature, and incubated with primary antibody at 4°C overnight. The primary antibodies were anti-VSVG (sc-365019, Santa Cruz biotechnology, United States), 1:1000 and anti-ICDH, 1:6000. The ICDH antisera were made in our previous study (Xu et al., 2010). The membrane was washed five times with TBST buffer (50 mM Tris, 150 mM NaCl, 0.05% Tween 20, pH 7.4), and incubated with 1:5,000 dilution of horseradish peroxidase conjugated secondary antibody (Shanghai Genomics) for 4 h at 4°C. Signals were detected using the ECL plus kit (GE Healthcare, Piscataway, NJ, United States) according to the manufacturer’s specified protocol.

### DNase I Footprinting Assay

DNase I footprinting assay was performed as described preciously (Zhao et al., 2017). Briefly, *hpaG1p* and T6SS4*p* were amplified by PCR with *hpaG1p-*FP-F/*hpaG1p-*FP-R and T6SS4*p-*FP-F/T6SS4*p-*FP-R primers, respectively. The PCR products were cloned into the pMD-18T vector (TaKaRa) as template to further amplify the fluorescent FAM labeled probes with primers M13R(FAM-labeled) and M13F(-47). The FAM-labeled probes were purified by the Wizard SV Gel and PCRClean-Up System (Promega) and quantified with NanoDrop 2000C (Thermo). For DNase I footprinting assay, 400 ng probes were incubated with various amount of His_6_-HpaR in 40 μl of each sample. After binding at 30°C for 30 min, 10 μl solution containing about 0.010 unit DNase I (Promega) and 100 nmol CaCl_2_ were added and further incubated for 1 min at 25°C. 140 μl DNase I stop solution (200 mM unbuffered sodium acetate, 30 mM EDTA and 0.15% SDS) was added into samples to stop the reaction. Samples were extracted by phenol/chloroform to remove proteins, precipitated with ethanol, washed and dissolved in 35 μl MiniQ water. The preparation of the DNA ladder, electrophoresis, and data analysis were similar to described previously (Wang et al., 2012).

### RNA-seq Experiments

*Yersinia pseudotuberculosis* and the Δ*hpaR* mutant were used for RNA-Seq transcriptomics analysis (Wang et al., 2017). Two strains (three biological replicates) were grown in YLB medium at 26°C to OD_600_ = 1.6. Total RNA was extracted from each sample for cDNA library construction by using bacteria total RNA isolation kit (Tiangen) and analyzed with the Bioanalyzer 2100 system (Agilent Technologies). Library sequencing was completed at the Beijing Genomic Institute (BGI-Shenzhen). The result of sequencing was aligned with the reference genome of *Y. pseudotuberculosis* and RPKM (Reads per kilobase transcriptome per million mapped reads) was used to normalize the expression level of genes. The differential expressed genes were shown as fold change calculated by log_2_ (RPKM of Δ*hpaR*/WT).

### Quantitative Real-Time PCR

All strains were cultured at mid-exponential, harvested, washed with PBS. Total RNA was isolated from each sample using the RNAprep Pure Cell/Bacteria Kit, treated with RNase-free DNase (Tiangen) and measured the purity and concentration by gel electrophoresis and spectrophotometer (NanoDrop, Thermo Scientific). 1.5 μg of total RNA was used to synthesis the first-strand cDNA by reverse transcriptase (TransGen Biotech). Quantitative real-time PCR (qRT-PCR) was performed in CFX96 Real-Time PCR Detection System (Bio-Rad) with TransStart Green qPCR SuperMix (TransGen Biotech). The qRT-PCR primers were listed in Supplementary Table S2 and qRT-PCR parameter was set as follows: 95°C for 30 s followed by 40 cycles of 94°C for 15 s, 52°C for 30 s. The relative abundance of 16S rRNA was used as the internal standard for standardization of results.

### Determination of Intracellular Ion Content

Intracellular ion content was determined as described previously (Wang et al., 2015). Briefly, *Y. pseudotuberculosis* wild-type, Δ*hpaR* and Δ*hpaR* (*hpaR*) strains were grown in YLB medium to mid-exponential. 20 ml culture were collected, washed with PBS, resuspended in 20 ml PBS buffer containing 0.4% glucose, 1.5 mM H_2_O_2_ and 1 μM Zn^2+^, and then incubated further for 20 min at 26°C. After centrifugation at low speed (4000 rpm) for 10 min, the supernatant was removed and the cell pellet weight was measured. Then the pellet was resuspended and chemically lysed on a rotating mixer at a slow setting for 20 min by using Bugbuster (Novagen, Madison, WI, United States) according to the manufacturer’s instructions. The protein concentration was analyzed with NanoDrop ND-1000 spectrophotometer (NanoDrop Technologies) according to the manufacturer’s instructions. The lysis solution was diluted 100-fold in 2% molecular grade nitric acid to 10 ml. These samples were analyzed by Inductively Coupled Plasma Mass Spectrometry (ICP-MS) (Varian 802-MS), and the results were corrected using the appropriate buffers for reference and dilution factors. Triplicate cultures of each strain were analyzed during a single experiment and the experiment was repeated at least three times.

### Biofilm Formation Assay

Biofilm formation was measured using the test tube, performed as previously described (Guan et al., 2015). *Y. pseudotuberculosis* strains were grown in YLB broth, and then transferred into 3 ml of M9 medium containing 0.4% glucose on a rotary shaker (220 rpm) at 26°C. After 24 h, the test tube was washed gently three times with PBS and stained with 1% crystal violet for 15 min, then washed three times with PBS again to remove redundant crystal violet. The remaining crystal violet was resolved with 95% ethanol and 200 μl of samples were measured the absorbance at 595 nm by a microplate reader (BioTek Instruments, Inc.).

### Cell Adhesion Assay

The adhesion ability of bacteria was measured as previously reported (Tan et al., 2017). HeLa cells were seeded into 12-well plate at a concentration of 1 × 10^5^ cells/ml before infection. *Y. pseudotuberculosis* wild-type, Δ*hpaR* and Δ*hpaR* (pKT100-*hpaR*) strains were grown at 26°C till OD_600_ = 1.0 and then collected, washed and resuspended in DMEM. The bacterial suspensions were serial diluted and plated onto YLB agar plates to count the actual number of the bacteria. HeLa cells were infected with bacterial strains at a multiplicity of infection (MOI) of 100 and then centrifuged for 5 min at 1500 rpm to promote contact between bacteria and HeLa cells. After 2 h, HeLa cells were washed for three times using prewarmed PBS. Cell-associated bacteria were liberated by treatment with 0.1% Triton X-100 in sterile PBS for 5 min. The number of cell-associated bacteria was divided by the total number of bacteria in a well to calculate the adhesion percentage.

### Mouse Infection

All mice were maintained and handled in accordance with the Regulations for the Administration of Affairs Concerning Experimental Animals approved by the State Council of People’s Republic of China. The protocol was approved by the Animal Welfare and Research Ethics Committee of Northwest A&F University (protocol number: NWAFUSM2018001). All strains growing in YLB broth at 26°C until mid-exponential were harvested, washed, resuspended in sterilized PBS to a final concentration of 3 × 10^9^ bacteria for survival assays. The 6–8 weeks old female C57BL/6 mice were orogastric infected with *Y. pseudotuberculosis* using a ball-tipped feeding needle. The survival rate of the mice was determined by monitoring the survival everyday for 24 days.

### Statistical Analysis

Statistical analyses of gene expression data, LacZ activity, survival assay, ROS determination, intracellular ion content determination, and cell adhesion assay were performed using paired two-tailed Student’s *t*-test. Statistical analyses were performed using GraphPad Prism software (GraphPad Software).

## Results

### Regulation of the *hpa* Gene Cluster in *Y*. *pseudotuberculosis*

**FIGURE 1:**
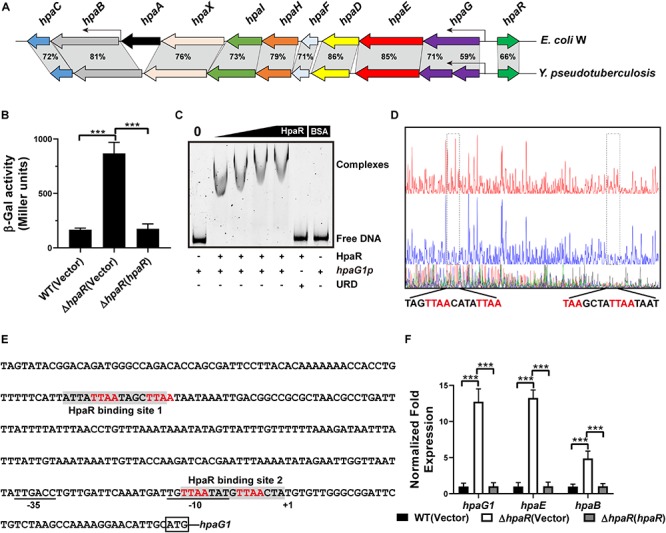
HpaR negatively regulates *hpa-meta* operon in *Yersinia pseudotuberculosis*. **(A)** Gene organization of *hpa-meta* cluster and identities of protein sequence in *Escherichia coli* W and *Y. pseudotuberculosis*. **(B)** β-Galactosidase activity of the *hpaG1* promoter in *Y. pseudotuberculosis* Wild-type, Δ*hpaR* mutant, and its complementary strain Δ*hpaR*(*hpaR*). Data shown are the average of three independent experiments. The error bars indicate standard deviations of the average. ***P < 0.001. **(C)** EMSA was performed to analyze the interactions between His_6_-HpaR and the *hpaG1* promoter. URD, unrelated DNA fragment. **(D)** Identification of the HpaR-binding site within the *hpaG1* promoter using the DNase I footprinting assay. Letters in red denote inverted repeat sequences. **(E)** The HpaR binding sites detected by DNase I footprinting assay were shown in the *hpaG1* promoter region by shading. Letters in red denote inverted repeat sequences. **(F)** Relative expression levels of *hpaG1*, *hpaE*, and *hpaB* were measured by quantitative RT-PCR in wild-type, Δ*hpaR* mutant, and complementary strain Δ*hpaR*(*hpaR*).

The 4-hydroxyphenylacetic acid (4-HPA) catabolic pathway was determined by alignment with *E*. *coli* W (ATCC 11105) while searching for the complete genome sequence of *Y*. *pseudotuberculosis* (Figure 1A). Analyses of the open reading frames of the gene cluster suggested that the 4-HPA metabolic cluster is composed of 11 genes in *Y*. *pseudotuberculosis*, similar to that of *E*. *coli* W (Roper et al., 1993; Prieto and Garcia, 1994). These genes include the genes of 4-HPA monooxygenase (*hpaBC*), the genes of homoprotocatechuate (HPC) *meta* cleavage enzymes (*hpaG1G2EDFHI*), the HPA transport protein gene (*hpaX*), and one regulatory gene (*hpaR*). All genes (*hpaG1G2EDFHIXBC*) lay in a continuous row in the same direction as in *E*. *coli*. The translation products of these genes were identified as the homologs of the 4-HPA hydroxylases in *E*. *coli* W with high identities (Figure 1A). HpaBC, the HPA monooxygenase, oxidizes 4-HPA or 3-HPA to HPC. HpaD (HPC 2,3-dioxygenase), HpaE (CHMS dehydrogenase), HpaF (CHM isomerase), HpaG (OPET decarboxylase), HpaH (hydratase), and *Hpa*I (HHED aldolase) form the HPA catabolic pathway, which degrades HPC in the pyruvic acid or succinic semialdehyde pathways. The HpaR protein shares 66, 66, 48, and 34% amino acid sequence identities with identified HpaR in *E*. *coli* C, *E*. *coli* W, *P. putida* U, and *B. xenovorans* LB400, respectively (Supplementary Figure S1). Thus, we propose the gene cluster from *ypk_2452* to *ypk_2462* as an *hpa-meta* gene cluster in *Y*. *pseudotuberculosis.*

In *E*. *coli* W, the *meta* cleavage enzymes (HpaGEDFHI) that catabolize 4-HPA are repressed by HpaR, and the HPA monooxygenase (HpaBC) is positively regulated by HpaA (Prieto and Garcia, 1997). To verify the role of HpaR in expression of the *hpa* gene cluster in *Y*. *pseudotuberculosis*, a single-copy *hpaG1p:lacZ* fusion reporter was introduced into the chromosomes of wild-type, Δ*hpaR* mutant, and complemented Δ*hpaR*(*hpaR*) strains. The β-galactosidase activity of the three strains was quantitatively measured (Figure 1B). The deletion of *hpaR* significantly increased the activity of the *hpa* promoter, which could be fully restored by introducing a complementary plasmid expressing HpaR (Figure 1B). Next, we analyzed the interaction between HpaR and the putative operator with an electrophoresis mobility shift assay (EMSA). Incubation of HpaR with a probe containing the promoter of the *hpa-meta* operon (*hpaG1p*) led to the formation of DNA–protein complexes (Figure 1C), which suggests direct interaction of HpaR and the *hpaG1p* promoter. DNase I footprinting analysis further revealed that the putative HpaR binding site was protected from digestion in DNA–HpaR complexes. Two HpaR-protected sites upstream of the transcriptional start site in the *hpaG1p* promoter were identified (Figures 1D,E). Thus, HpaR specifically recognizes an operator within the *hpa-meta* promoter, likely influencing its expression. Indeed, negative regulation of the *hpa-meta* operon by HpaR was confirmed by qRT-PCR analyses. The data indicated that the expression of *hpaG1*, *hpaE*, and *hpaB* was upregulated in Δ*hpaR*, and such an increase could be restored to wild-type levels by complementation with the expression plasmid pKT100-*hpaR* (Figure 1F). Overall, in *Y*. *pseudotuberculosis*, HpaR functions as a repressor and negatively regulates the *hpa-meta* gene cluster in a similar manner to *E*. *coli* W.

### Genome-Wide Analysis of the Genes Regulated by HpaR

The multiple antibiotic resistance regulator (MarR) family transcriptional regulator has a variety of biological functions, including resistance to multiple antibiotics and other toxic chemicals such as organic solvents, household disinfectants, and oxidative stress agents (Alekshun and Levy, 1999; Wei et al., 2007). As a MarR family regulator, we speculate that HpaR may play other regulatory roles besides aromatic degradation. Thus, RNA sequencing (RNA-seq) was employed to detect the genes regulated by HpaR, except the *hpa-meta* gene cluster.

**FIGURE 2:**
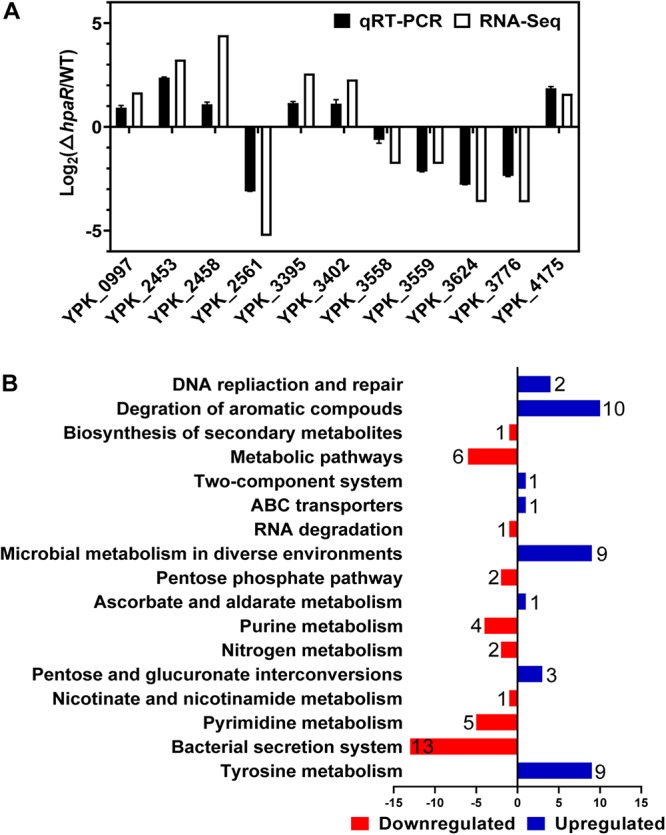
Transcriptomics analysis of HpaR regulated genes in *Y. pseudotuberculosis*. **(A)** Validation of RNA-Seq data using qRT-PCR. Eleven representative genes were evaluated for validation of the RNA-seq data using qRT-PCR. **(B)** KEGG pathway analysis of differentially expressed genes (*hpaR* mutant VS wild-type). The red and blue bars represent up- and down-regulated genes, respectively, and the numeric labels represent the number of genes with corresponding function.

To identify the HpaR-dependent expression genes in *Y*. *pseudotuberculosis*, RNA was extracted for RNA-seq from both wild-type and Δ*hpaR* mutant strains in mid-exponential phase (SRA accession: PRJNA612340). After RNA-seq, quality control and gene expression level analysis were performed by fragments per kilobase of transcript per million mapped reads (FPKM). A total of 106 gene candidates that exhibited differential expression were found between wild-type and Δ*hpaR* mutant strains, including 57 upregulated and 49 downregulated genes (Supplementary Table S3). To verify the RNA-seq data, qRT-PCR analysis of 11 representative genes was conducted. The *log*_2_-transformed values of each gene were in good agreement with the RNA-seq data (Figure 2A), which confirmed the credibility of the RNA-seq data. Subsequently, the functions of differentially expressed genes were identified by KEGG pathway analysis. As shown in Figure 2B, 17 different pathways were found in the DGE (Different Gene Expression), in which eight pathways were up-regulated and nine were down-regulated. These included degradation of aromatic compounds, metabolism, microbial metabolism in a diverse environment, purine metabolism, bacterial secretion system, and tyrosine metabolism. In the RNA-seq data, two gene clusters were found to be fully regulated by HpaR. One cluster was the *hpa-meta* operon, which was 2.3–4.56-fold upregulated in the Δ*hpaR* mutant (Table 1). The other was the T6SS4 gene cluster in *Y*. *pseudotuberculosis.* T6SS is a newly described secretion system in many Gram-negative bacteria (Bingle et al., 2008; Jani and Cotter, 2010). The RNA-seq data revealed that the genes in the T6SS4 operon were 0.54–0.96-fold downregulated in the Δ*hpaR* mutant (Table 2). Hemolysin co-regulated protein (Hcp), which forms the hexameric rings for the T6SS syringe needle, was 0.68-fold downregulated. Valine-glycine repeat protein G (VgrG) was 0.54-fold downregulated. The periplasmic domain protein IcmF was 0.62-fold downregulated. ImpA/B (VipA/VipB), which serve as tail sheath proteins in T6SS, were 0.73/0.94-fold downregulated. The same was true with other structural genes. These data suggest that the *hpa-meta* operon was negatively regulated by HpaR, consistent with the transcription of chromosomal *hpaG1p:lacZ* fusions. The data also suggest that T6SS4 is positively regulated by the transcriptional regulator HpaR.

**TABLE 1:** The *hpa-meta* operon genes regulated by HpaR in *Y. pseudotuberculosis.*

**Locus tag**	**Product**	**Gene description**	Fold change*
YPK_2452	HpaC	4-Hydroxyphenylacetate-3-monooxygenase small chain	2.31
YPK_2453	HpaB	4-Hydroxyphenylacetate-3-monooxygenase large chain	3.24
YPK_2454	HpaX	4-Hydroxyphenylacetate permease	3.37
YPK_2455	HpaI	2,4-Dihydroxyhept-2-ene-1,7-dioic acid aldolase	3.83
YPK_2456	HpaH	2-Oxo-hept-3-ene-1,7-dioate hydratase	3.91
YPK_2457	HpaF	5-Carboxymethyl-2-hydroxymuconate isomerase	4.25
YPK_2458	HpaD	3,4-Dihydroxyphenylacetate 2,3-dioxygenase	4.42
YPK_2459	HpaE	5-Carboxymethyl-2-hydroxymuconic-semialdehyde dehydrogenase	4.45
YPK_2460	HpaG2	2-Hydroxyhepta-2,4-diene-1,7-dioate isomerase	4.56
YPK_2461	HpaG1	2-Hydroxyhepta-2,4-diene-1,7-dioate isomerase	4.50

*Fold change: log_2_(Δ*hpaR*/WT).

**TABLE 2:** T6SS4 genes regulated by HpaR in *Y. pseudotuberculosis.*

**Locus tag**	**Product**	**Gene description**	Fold change*
YPK_3550	ImpL	Type VI secretion system protein ImpL	−1.52
YPK_3551	ImpK	Type VI secretion system protein ImpK	−1.53
YPK_3552	ImpJ	Type VI secretion system protein ImpJ	−1.63
YPK_3556		Pentapeptide repeat protein	−1.74
YPK_3557		Pentapeptide repeat protein	−1.77
YPK_3558	VgrG	Type VI secretion system secreted protein VgrG	−1.78
YPK_3559	VasG	Type VI secretion system protein VasG	−1.70
YPK_3560	ImpH	Type VI secretion system protein ImpH	−1.52
YPK_3561	ImpG	Type VI secretion system protein ImpG	−1.79
YPK_3564	ImpC	Type VI secretion system protein ImpC	−1.77
YPK_3565	ImpB	Type VI secretion system protein ImpB	−1.69
YPK_3566	ImpA	Type VI secretion system protein ImpA	−1.90

*Fold change: log_2_(Δ*hpaR*/WT).

### HpaR Positively Regulates T6SS4 Expression by Directly Binding to Its Promoter

**FIGURE 3:**
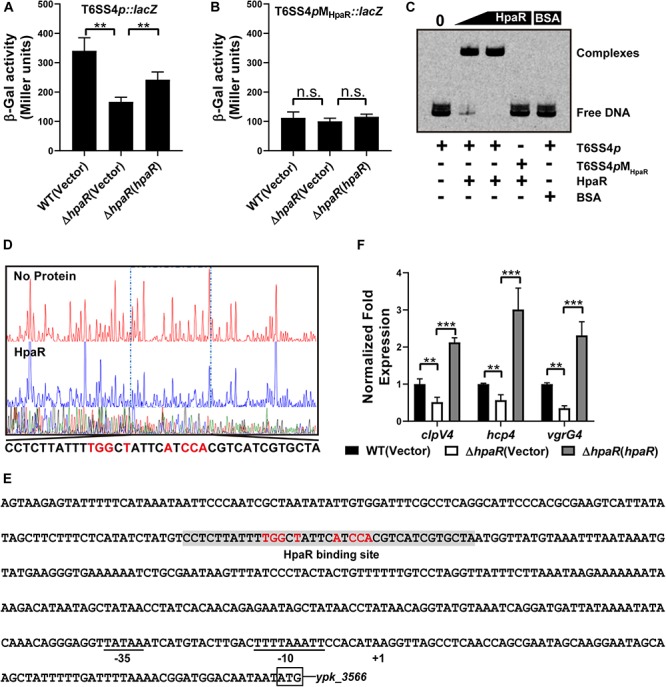
HpaR positively regulates the T6SS4 expression in *Y. pseudotuberculosis*. β-galactosidase activity of the T6SS4 promoter **(A)** and T6SS4 promoter with mutations in the HpaR binding site **(B)** in *Y. pseudotuberculosis* Wild-type, Δ*hpaR* mutant, and its complementary strain Δ*hpaR*(*hpaR*). Data shown are the average of three independent experiments. The error bars indicate standard deviations of the average. **P < 0.01; n.s., not significant. **(C)** EMSA was performed to analyze the interactions between His_6_-HpaR and the T6SS4 promoter (T6SS4*p*) or the promoter mutant identified His_6_-HpaR binding region (T6SS4*p*M_*HpaR*_). **(D)** Identification of the HpaR-binding site within the T6SS4 promoter using the DNase I footprinting assay. Letters in red denote inverted repeat sequences. **(E)** The HpaR binding site detected by DNase I footprinting assay were shown in the T6SS4 promoter region by shading. Letters in red denote inverted repeat sequences. **(F)** Relative expression levels of *clpV4*, *hcp4*, and *vgrG4* were measured by qRT-PCR. The error bars indicate the standard deviation from three independent experiments. ***P < 0.001; **P < 0.01.

Based on the RNA-seq data, we found HpaR could positively regulate the expression of T6SS4. To further determine the role of HpaR in the expression of T6SS4, a single copy T6SS4*p:lacZ* fusion was introduced into the chromosomes of wild-type, *hpaR* mutant, and complemented Δ*hpaR*(*hpaR*) strains and LacZ activity of the resulting strains was quantitatively measured (Figure 3A). Compared to the wild-type strain, the T6SS4*p:lacZ* fusion was decreased significantly in Δ*hpaR*; this decrease could be restored by the complementary plasmid (pKT100-*hpaR*). This suggests that HpaR positively regulates T6SS4 expression. To test whether the T6SS4 gene clusters were regulated by HpaR directly, the interaction between HpaR and the T6SS4 promoter was examined by EMSA. Incubation of a probe containing the T6SS4 promoter with HpaR led to the formation of DNA–protein complexes (Figure 3C). DNase I footprinting analysis revealed that the binding site was protected from digestion in DNA–HpaR complexes, further indicating the recognition of this DNA element by HpaR (Figure 3D). An HpaR-protected site (CCTCTTATTTTGGCTATTCATCCACGTCATCGTGCTA) upstream of the -35 and -10 elements was identified (Figure 3E).

The interactions between HpaR and the T6SS4 promoter appeared to be site-specific, as mutation of the HpaR binding site in the T6SS4 promoter prevented formation of the protein–DNA complex (Figure 3C) and abolished HpaR dependent T6SS4 regulation (Figure 3B). The positive regulation of T6SS4 by HpaR was further confirmed by qRT-PCR analysis (Figure 3F). The data indicated that the expression of T6SS4 components *clpV4*, *hcp4*, and *vgrG4* were reduced in Δ*hpaR*, which could be restored by plasmid (pKT100-*hpaR*). Thus, HpaR recognizes and binds to the promoter of T6SS4 specifically to activate expression.

### HpaR Affects the Antioxidant Activity of *Y*. *pseudotuberculosis* by Regulating T6SS4

**FIGURE 4:**
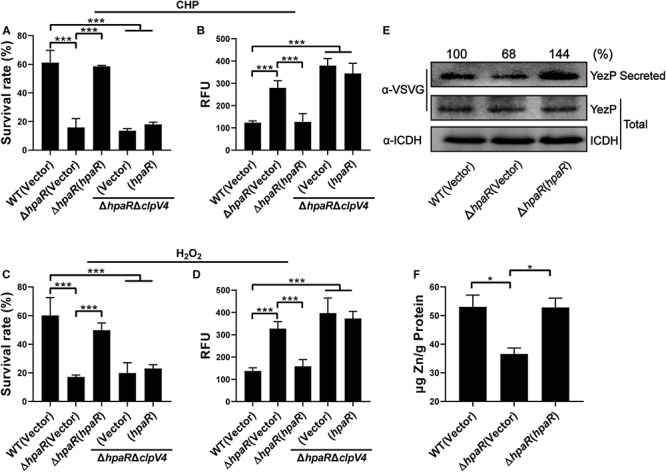
HpaR plays a role in resisting oxidative stress via T6SS4. Survival rate of indicated *Y. pseudotuberculosis* strains were determined after treatment with CHP **(A)** or H_2_O_2_ **(C)** for 1 h. **(B,D)** Intracellular ROS of indicated strains were stained with H_2_DCFDA dye (Invitrogen) and fluorescence signals were measured using a SpectraMax M2 Plate Reader (Molecular Devices) with excitation/emission wavelengths of 495/520. **(E)** Western blot detection of YezP secretion in *Y. pseudotuberculosis* wild-type, Δ*hpaR* mutant, and its complementary strain Δ*hpaR*(*hpaR*). Proteins in culture supernatant of relevant *Y. pseudotuberculosis* strains expressing YezP-VSVG were probed for VSVG (upper) by immunoblotting. **(F)** Mid-exponential phase of *Y. pseudotuberculosis* strains were exposed to 1.5 mM H_2_O_2_ for 30 min in PBS containing 1 μM ZnCl_2_. Zn^2+^ associated with bacterial cells was measured by inductively coupled plasma mass spectrometry (ICP-MS). Data shown are the average of three independent experiments. The error bars indicate the standard deviation from three independent experiments. ***P < 0.001; **P < 0.01; *P < 0.05.

In our previous study, T6SS4 of *Y*. *pseudotuberculosis* was discovered to play important roles in multiple stress defenses, especially oxidative stress (Wang et al., 2015). The regulation of T6SS4 by HpaR prompted us to examine whether HpaR plays a role in oxidative stress resistance via T6SS4. To test the hypothesis that HpaR exerts antioxidant activity by regulating T6SS4, the survival rate and ROS level in cells treated with cumene hydroperoxide (CHP) or hydrogen peroxide (H_2_O_2_) were measured. After incubation with CHP, approximately 60% of wild-type cells survived. The survival rate of the mutant lacking *hpaR* decreased to 16%, and a 13% survival rate was observed for the double mutant Δ*hpaR*Δ*clpV4*. Expressing HpaR could fully rescue such a decrease in the Δ*hpaR*, but only increased the survival rate slightly in the double mutant Δ*hpaR*Δ*clpV4* (Figure 4A). The ROS level under oxidative stress was also examined using CM-H_2_DCFDA fluorescent dye. The data showed that the ROS level in Δ*hpaR* and Δ*hpaR*Δ*clpV4* was higher than the wild-type strain. Complementation with the plasmid pKT100-*hpaR* could reduce the ROS level in Δ*hpaR*, but not in the Δ*hpaR*Δ*clpv4* double mutant (Figure 4B). Similar results were obtained with H_2_O_2_ treatment (Figures 4C,D). T6SS4 could secrete the zinc-binding protein YezP for zinc import in *Y*. *pseudotuberculosis* (Wang et al., 2015). Thus, YezP-VSVG was expressed in relevant *Y*. *pseudotuberculosis* strains to test the secretion of YezP. Significant amounts of YezP-VSVG could be readily detected in culture supernatant from wild-type bacteria. The secretion of YezP decreased by 32% in the Δ*hpaR* mutant and was completely restored in a mutant complemented with HpaR (Figure 4E). The zinc contents in bacteria challenged with H_2_O_2_ were measured using inductively coupled plasma mass spectrometry (ICP-MS). The results revealed that the deletion of *hpaR* significantly lowered the intracellular Zn^2+^ level and expression of HpaR restored the defects (Figure 4F). These results established that HpaR benefited *Y*. *pseudotuberculosis* resisting oxidative stress by upregulating T6SS4.

### HpaR Contributes to Biofilm Formation and Adhesion to Epithelial Cells

**FIGURE 5:**
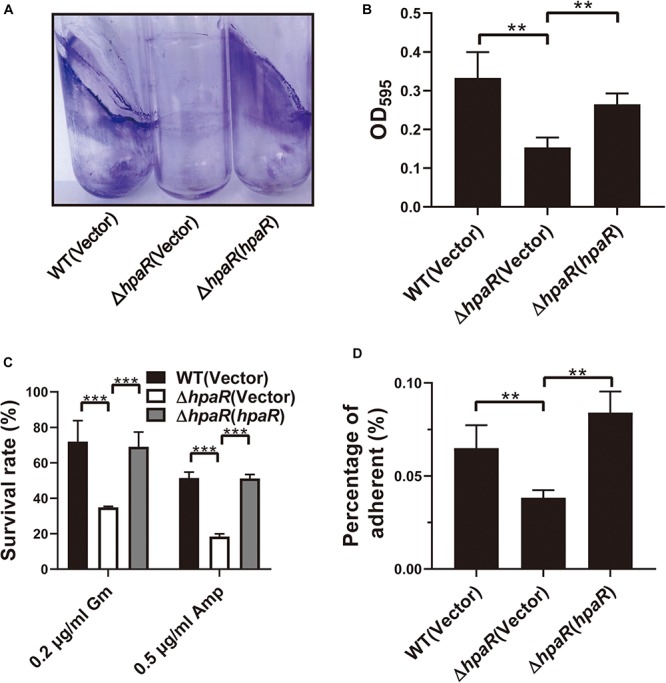
HpaR is involved in biofilm formation, antibiotic resistance and adhesion to eukaryotic cells. **(A)** Direct observation of *Y. pseudotuberculosis* biofilm formation by Wild-type, Δ*hpaR* mutant, and its complementary strain Δ*hpaR* (*hpaR*). The results shown the biofilm biomass stained with crystal violet in glass test tubes. **(B)** Quantitative measurement of biofilm formation by resolving in 95% ethanol and measuring the absorbance at OD_595_. **(C)** The survival rate of Wild-type, Δ*hpaR* mutant, and its complementary strain Δ*hpaR* (*hpaR*) under 0.2 μg/ml gentamicin (Gm) or 0.5 μg/ml ampicillin (Amp) treatment. **(D)** Adhesion of the strains to HeLa cells. Adherence level is expressed as the percentage of adherent bacteria relative to the inoculum count. Data shown are the average of three independent experiments; the error bars indicate the standard deviation from three independent experiments. ***P < 0.001; **P < 0.01; *P < 0.05; n.s., not significant.

Similar to other Gram-negative bacteria, *Y*. *pseudotuberculosis* is capable of forming biofilms on abiotic surfaces. The biofilm formation ability of wild-type, Δ*hpaR* mutant, and complemented Δ*hpaR*(*hpaR*) strains were observed in a test tube by staining with crystal violet (Figure 5A) and quantified by resolving with 95% ethanol and measuring the absorbance at 595 nm (Figure 5B). The Δ*hpaR* mutant showed attenuated biofilm formation when compared to wild-type and the decrease could be restored by expressing *hpaR*. Note that there’s no significant difference between the growth activities of these strains (Supplementary Figure S2). Biofilm formation plays an important role in the pathogen lifecycle. It is imperative in chronic infection, antibiotic resistance, and adhesion to host cells. Thus, ampicillin and gentamicin, two commonly used antibiotics in clinics, were used to verify viability of the *hpaR* mutant in antibiotic resistance. The survival rates of the Δ*hpaR* strain were only 34 and 18% following gentamicin and ampicillin treatment, respectively, while approximately 72 and 51% of wild-type cells survived under the same conditions (Figure 5C). This suggests that the Δ*hpaR* mutant was more sensitive to antibiotics than the wild-type. Adhesion to host cells is a crucial step in the infection process and host colonization. Thus, the adherence of wild-type, *hpaR* mutant, and complemented strains to HeLa cells was evaluated. As shown in Figure 5D, the Δ*hpaR* strain had fewer adherences to HeLa cells than wild-type. Overall, *hpaR* may assist in the maintenance of biofilm formation activity, antibiotic resistance, and adhesion to eukaryotic cells.

### *Y*. *pseudotuberculosis* Mutant Lacking *hpaR* Is Defective in Virulence in Mice

**FIGURE 6:**
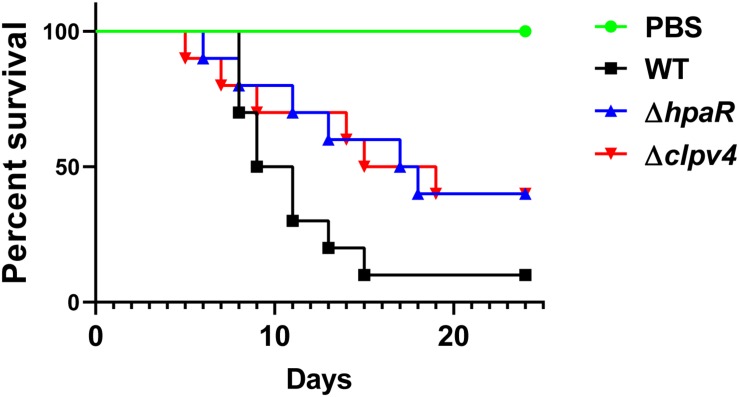
Yptb mutants lacking *hpaR* or T6SS4 are defective in virulence against mice. Bacterial strains grown in YLB were washed twice in sterilized PBS and used for orogastric infection of 6–8 weeks old female C57BL/6 mice using a ball-tipped feeding needle. For survival assays 3 × 10^9^ bacteria of each strain were applied to different groups of mice (*n* = 10/strain), and the survival rate of the mice was determined by monitoring the survival daily for 24 days.

Biofilm formation is helpful for the survival and colonization of *Y*. *pseudotuberculosis* in hosts. In addition, oxidative burst is an important microbial-killing mechanism in phagocytes. The findings that HpaR affects the antioxidant activity, biofilm formation and the ability of adhesion to eukaryotic cells point to its role in the virulence of *Y*. *pseudotuberculosis.* To examine this, C57BL/6 mice were inoculated orogastrically with relevant bacterial strains. Wild-type bacteria caused more than 90% lethality within 3 weeks of inoculation. By contrast, consistent with the hypothesis, mice infected with mutants lacking *hpaR* or T6SS4 had better survival rates than the wild-type (Figure 6). The data indicate that *hpaR* affects the virulence of *Y*. *pseudotuberculosis* in the infection of mammalian hosts.

## Discussion

MarR family transcriptional regulators are widely distributed in bacteria and archaea (Perez-Rueda and Collado-Vides, 2001; Perez-Rueda et al., 2004). They play crucial roles in the bacterial lifecycle as they control various genes involved in antibiotic resistance, aromatic compound catabolism, oxidative stress responses, and produce of virulence factors (Alekshun and Levy, 1999; Fiorentino et al., 2007; Deochand and Grove, 2017). Some of these regulators are involved in the degradation of specific aromatic compounds (Diaz and Prieto, 2000). For example, BadR in *Rhodopseudomonas palustris* regulates anaerobic benzoate degradation in cooperation with AadR (Egland and Harwood, 1999). In *Comamonas testosteroni* BR60, CbaR controls the *cbaABC* operon for converting 3-chlorobenzoate to protocatechuate (PCA) and 5-Cl-PCA (Providenti and Wyndham, 2001). HpaR was first identified in *E*. *coli* W (Roper et al., 1993). As a repressor, HpaR regulates the *hpa-meta* operon in 4-HPA catabolism (Prieto et al., 1996; Galan et al., 2003). However, whether HpaR has other functions remains unclear. Here, we report that in *Y. pseudotuberculosis*, HpaR acts as a dual-functional regulator. It not only negatively regulates the *hpa-meta* cluster but also positively controls T6SS4 expression to resist oxidative stress, which maintains cellular ROS levels.

The *hpa-meta* cluster in *E*. *coli* W is composed of 11 genes within two operons, *hpaBC* and *hpaGEDFHI*, which are controlled by HpaA and HpaR, respectively (Galan et al., 2001). Based on sequence alignment with *E*. *coli* W, we identified the *hpa-meta* cluster in *Y*. *pseudotuberculosis*. An HpaA homolog was not identified in *Y*. *pseudotuberculosis*. The *hpa-meta* cluster of one operon consisted of 11 genes, *hpaG1G2EDFHIXBC*, and one regulator, HpaR (Figure 1A). Using a β-galactosidase activity assay and qRT-PCR, we revealed that HpaR represses expression of the *hpaG1G2EDFHIXBC* operon in *Y*. *pseudotuberculosis*, similar to *E*. *coli* W. HpaR binding sites 1 and 2, both composed of a 15 bp region, were identified on the *hpaG1* promoter containing a palindromic sequence of 4 bp (TTAA-XXXX-TTAA) on each side separated by 4 bp similar to *E*. *coli* W (Figure 1E). Site 1 was located -189 bp relative to the transcription start site + 1 of the *hpaG1* promote. Site 2 was centered at position -8 of the *hpaG1* promoter, overlapping the -10 element, which implies that HpaR inhibits the binding of RNA polymerase on the promoter to repress transcription of the *hpa-meta* cluster.

So far HpaR was specifically recognized as a repressor of the 4-HPA catabolic pathway (Galan et al., 2003; Arcos et al., 2010; Mendez et al., 2011). Unexpectedly, in the RNA-seq based transcriptomics analysis we found that in addition to the *hpa-meta* cluster genes, genes of the T6SS4 cluster were also found to be regulated by HpaR. Based on the β-galactosidase activity assay and qRT-PCR results, we confirmed that HpaR acts as an activator rather than a repressor to regulate T6SS4 (Figure 3A). We also identified the HpaR binding site on the T6SS4 promoter located -241 bp relative to the transcription start site + 1 of the T6SS4 promote, containing an imperfect invert sequence TGGXT-XXXX-AXCCA on each side separated by 4 bp (Figure 3E). The HpaR binding site of *hpaG1* and T6SS4 promoter both contain a 4–5 bp palindromic sequence on each side separated by 4 bp but the site on T6SS4 promoter is imperfect. The binding site of T6SS4 promoter is far from the transcription start site. Besides, the GC content of binding site on T6SS4 promoter is higher than that on *hpaG1* promoter. We presume that HpaR may bind to the stable site upstream of the -35 and -10 elements recruiting RNA polymerase to activate the transcription of T6SS4.

T6SS has been recognized as an anti-bacterial weapon to attack target cells in a contact-dependent manner (Durand et al., 2014; Ho et al., 2014; Russell et al., 2014). However, our previous report showed that the T6SS4 in *Y*. *pseudotuberculosis* is related to resistance to multiple environmental stresses, including high osmolarity, low pH, and oxidative stress (Song et al., 2015; Wang et al., 2015). We therefore speculated that HpaR might protect against oxidative stress via T6SS4. And we confirmed this by observing the survival rate and determining the intracellular ROS levels of wild-type and mutant strains under oxidative stress (Figures 4A–D). *Y*. *pseudotuberculosis* could import Zn^2+^ via secretion of YezP through T6SS4 to maintain intracellular ROS levels (Wang et al., 2015). Our results showed that the levels of YezP secreted by T6SS4 and the intracellular Zn^2+^ levels of Δ*hpaR* were also decreased under H_2_O_2_ stress (Figures 4E,F), indicating that HpaR regulates the expression of T6SS4 to resist oxidative stress.

Biofilm formation was also affected by HpaR (Figures 5A,B). However, no other biofilm-related gene was found to be differentially expressed in the RNA-seq results. Biofilm formation is complex—it may be indirectly regulated by other mechanisms that will require further investigation. Biofilm formation is beneficial to bacterial survival in a harmful environment and is associated with adhesion to eukaryotic cells (Flemming and Wingender, 2010; Hall and Mah, 2017). Our results also showed that HpaR was involved in antibiotic resistance and adhered to HeLa cells (Figures 5C,D). *Y*. *pseudotuberculosis* is an enteric pathogen of animals and humans (Smego et al., 1999). In host cells, the oxidative burst is an important method to kill bacteria and biofilm formation is essential for bacterial infection (Flemming and Wingender, 2010; Slauch, 2011). Besides, the adhesion to host cell is also crucial for bacterial colonization. It has been shown that T6SS4 is required for *Y*. *pseudotuberculosis* virulence in a mouse model (Wang et al., 2015). This work revealed that HpaR also plays important roles in virulence and biofilm formation, potentially also via regulation of T6SS4.

**FIGURE 7:**
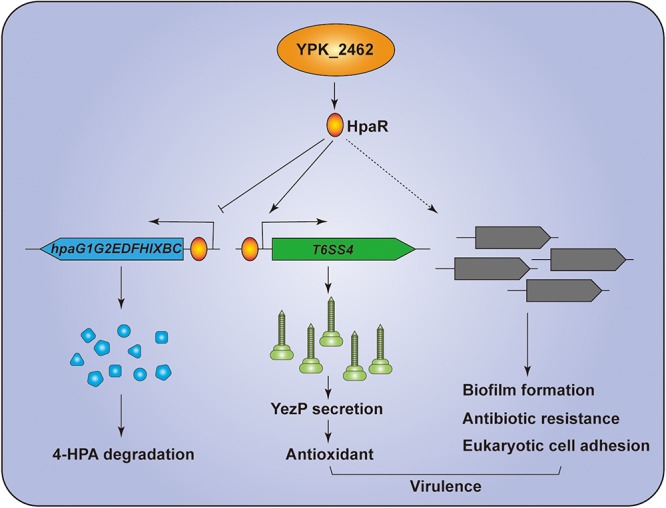
Hypothetical model for the function of HpaR in *Y. pseudotuberculosis*. HpaR represses the 4-HPA catabolic pathway but activates the transcription of T6SS4 to maintain ROS levels in response to oxidative stress. HpaR also affects the biofilm formation, eukaryotic cell adhension, antibiotic resistance, and virulence of *Y. pseudotuberculosis*.

In summary, we revealed that HpaR is a dual-functional regulator in *Y*. *pseudotuberculosis*. HpaR not only represses expression of genes of the 4-HPA catabolic pathway but also activates the transcription of T6SS4 to acquire Zn^2+^ to maintain ROS levels in response to oxidative stress. Biofilm formation, antibiotic resistance, eukaryotic cell adhesion, and bacterial virulence were also affected by *hpaR* (Figure 7). This study has greatly expanded the function of HpaR beyond the regulation of aromatic compound catabolism and revealed a new pathway to regulate T6SS4 in response to environmental stress.

## Data Availability Statement

The datasets generated for this study can be found in the RNA-seq data was deposited in SRA (SRA accession: PRJNA612340).

## Ethics Statement

All mice were maintained and handled in accordance with the Regulations for the Administration of Affairs Concerning Experimental Animals approved by the State Council of People’s Republic of China. The protocol was approved by the Animal Welfare and Research Ethics Committee of Northwest A&F University (protocol number: NWAFUSM2018001).

## Author Contributions

ZW, TW, YY, and XS conceived the study, designed the experiments, analyzed the data, and wrote the manuscript. ZW, TW, RC, ZZ, KC, ML, YH, HG, LX, and YW performed the experimental work.

## Conflict of Interest

The authors declare that the research was conducted in the absence of any commercial or financial relationships that could be construed as a potential conflict of interest.

## Footnotes

1 https://www.ncbi.nlm.nih.gov/

2 http://espript.ibcp.fr/ESPript/cgi-bin/ESPript.cgi

## Supplementary Material

FIGURE S1 Protein sequence alignment of HpaR homologs by CLUSTAL W. The sequences used in alignment have been deposited in the GenBank database [Accession No. EcC *Escherichia coli* C (S56952.1), EcW *Escherichia coli* W (Z37980.2), Yptb *Yersinia pseudotuberculosis* YPIII (ACA68739.1), PpU *Pseudomonas putida* U (FJ904934.1), BxLB400 *Burkholderia xenovorans* LB400 (ABE33958.1)]. The result was exported by ESPript (http://espript.ibcp.fr/ESPript/cgi-bin/ESPript.cgi).

FIGURE S2 Growth curves of the wild-type (WT), Δ*hpaR* mutant, and the complemented strain Δ*hpaR* (*hpaR*) under normal condition. The growth of the indicated strains in YLB medium was monitored by measuring OD_600_ at indicated time points under 26°C.

TABLE S1 The strains and plasmids used in this study.

TABLE S2 Primers used in this study.

TABLE S3 Genes differentially transcribed in Δ*hpaR* mutant compared to the *Y. pseudotuberculosis* wild-type detected by RNA-seq.

## References

[B1] Alekshun M. N. Levy S. B. (1999). The mar regulon: multiple resistance to antibiotics and other toxic chemicals. *Trends Microbiol.* 7 410–413. 10.1016/s0966-842x(99)01589-1589 10498949

[B2] Alekshun M. N. Levy S. B. Mealy T. R. Seaton B. A. Head J. F. (2001). The crystal structure of MarR, a regulator of multiple antibiotic resistance, at 2.3 A resolution. *Nat. Struct. Biol.* 8 710–714. 10.1038/90429 11473263

[B3] Arcos M. Olivera E. R. Arias S. Naharro G. Luengo J. M. (2010). The 3,4-dihydroxyphenylacetic acid catabolon, a catabolic unit for degradation of biogenic amines tyramine and dopamine in *Pseudomonas putida* U. *Environ. Microbiol.* 12 1684–1704. 10.1111/j.1462-2920.2010.02233.x 20482587

[B4] Barbour M. G. Bayly R. C. (1981). Control of meta-cleavage degradation of 4-hydroxyphenylacetate in *Pseudomonas putida*. *J. Bacteriol.* 147 844–850. 10.1128/jb.147.3.844-850.1981 6895079 PMC216120

[B5] Basler M. (2015). Type VI secretion system: secretion by a contractile nanomachine. *Philos. Trans. R. Soc. Lond. B Biol. Sci.* 370 1679 10.1098/rstb.2015.0021 PMC4632598 26370934

[B6] Bingle L. E. Bailey C. M. Pallen M. J. (2008). Type VI secretion: a beginner’s guide. *Curr. Opin. Microbiol.* 11 3–8. 10.1016/j.mib.2008.01.006 18289922

[B7] Brubaker R. R. (1991). Factors promoting acute and chronic diseases caused by yersiniae. *Clin. Microbiol. Rev.* 4 309–324. 10.1128/cmr.4.3.309 1889045 PMC358201

[B8] Cooper R. A. Skinner M. A. (1980). Catabolism of 3- and 4-hydroxyphenylacetate by the 3,4-dihydroxyphenylacetate pathway in *Escherichia coli*. *J. Bacteriol.* 143 302–306. 10.1128/jb.143.1.302-306.1980 6995433 PMC294232

[B9] D’Autreaux B. Toledano M. B. (2007). ROS as signalling molecules: mechanisms that generate specificity in ROS homeostasis. *Nat. Rev. Mol. Cell Biol.* 8 813–824. 10.1038/nrm2256 17848967

[B10] Deochand D. K. Grove A. (2017). MarR family transcription factors: dynamic variations on a common scaffold. *Crit. Rev. Biochem. Mol. Biol* 52 595–613. 10.1080/10409238.2017.1344612 28670937

[B11] Diaz E. Prieto M. A. (2000). Bacterial promoters triggering biodegradation of aromatic pollutants. *Curr. Opin. Biotechnol.* 11 467–475. 10.1016/s0958-1669(00)00126-129 11024365

[B12] Durand E. Cambillau C. Cascales E. Journet L. (2014). VgrG, Tae, Tle, and beyond: the versatile arsenal of Type VI secretion effectors. *Trends Microbiol.* 22 498–507. 10.1016/j.tim.2014.06.004 25042941

[B13] Egland P. G. Harwood C. S. (1999). BadR, a new MarR family member, regulates anaerobic benzoate degradation by Rhodo *Pseudomonas palustris* in concert with AadR, an Fnr family member. *J. Bacteriol.* 181 2102–2109. 10.1128/jb.181.7.2102-2109.1999 10094687 PMC93622

[B14] Fallman M. Gustavsson A. (2005). Cellular mechanisms of bacterial internalization counteracted by Yersinia. *Int. Rev. Cytol.* 246 135–188. 10.1016/S0074-7696(05)46004-46000 16164968

[B15] Fiorentino G. Ronca R. Cannio R. Rossi M. Bartolucci S. (2007). MarR-like transcriptional regulator involved in detoxification of aromatic compounds in *Sulfolobus solfataricus*. *J. Bacteriol.* 189 7351–7360. 10.1128/JB.00885-887 17675388 PMC2168448

[B16] Flemming H. C. Wingender J. (2010). The biofilm matrix. *Nat. Rev. Microbiol.* 8 623–633. 10.1038/nrmicro2415 20676145

[B17] Galan B. Diaz E. Prieto M. A. Garcia J. L. (2000). Functional analysis of the small component of the 4-hydroxyphenylacetate 3-monooxygenase of *Escherichia coli* W: a prototype of a new Flavin:NAD(P)H reductase subfamily. *J. Bacteriol.* 182 627–636. 10.1128/jb.182.3.627-636.2000 10633095 PMC94324

[B18] Galan B. Kolb A. Garcia J. L. Prieto M. A. (2001). Superimposed levels of regulation of the 4-hydroxyphenylacetate catabolic pathway in *Escherichia coli*. *J. Biol. Chem.* 276 37060–37068. 10.1074/jbc.M103033200 11477101

[B19] Galan B. Kolb A. Sanz J. M. Garcia J. L. Prieto M. A. (2003). Molecular determinants of the hpa regulatory system of *Escherichia coli*: the HpaR repressor. *Nucleic Acids Res.* 31 6598–6609. 10.1093/nar/gkg851 14602920 PMC275547

[B20] Green E. R. Clark S. Crimmins G. T. Mack M. Kumamoto C. A. Mecsas J. (2016). Fis is essential for yersinia pseudotuberculosis virulence and protects against reactive oxygen species produced by phagocytic cells during infection. *PLoS Pathog.* 12:e1005898 10.1371/journal.ppat.1005898 PMC5045184 27689357

[B21] Grove A. (2013). MarR family transcription factors. *Curr. Biol.* 23 R142–R143. 10.1016/j.cub.2013.01.013 23428319

[B22] Guan J. Xiao X. Xu S. Gao F. Wang J. Wang T. (2015). Roles of RpoS in *Yersinia pseudotuberculosis* stress survival, motility, biofilm formation and type VI secretion system expression. *J. Microbiol.* 53 633–642. 10.1007/s12275-015-0099-96 26310305

[B23] Hall C. W. Mah T. F. (2017). Molecular mechanisms of biofilm-based antibiotic resistance and tolerance in pathogenic bacteria. *FEMS Microbiol. Rev.* 41 276–301. 10.1093/femsre/fux010 28369412

[B24] Ho B. T. Dong T. G. Mekalanos J. J. (2014). A view to a kill: the bacterial type VI secretion system. *Cell Host Microb.* 15 9–21. 10.1016/j.chom.2013.11.008 PMC3936019 24332978

[B25] Huang Y. Zhao K. X. Shen X. H. Jiang C. Y. Liu S. J. (2008). Genetic and biochemical characterization of a 4-hydroxybenzoate hydroxylase from *Corynebacterium glutamicum*. *Appl. Microbiol. Biotechnol.* 78 75–83. 10.1007/s00253-007-1286-1280 18071645

[B26] Jani A. J. Cotter P. A. (2010). Type VI secretion: not just for pathogenesis anymore. *Cell Host Microb.* 8 2–6. 10.1016/j.chom.2010.06.012 PMC2913581 20638635

[B27] Lin J. Zhang W. Cheng J. Yang X. Zhu K. Wang Y. (2017). A *Pseudomonas* T6SS effector recruits PQS-containing outer membrane vesicles for iron acquisition. *Nat. Commun.* 8:14888 10.1038/ncomms14888 PMC5379069 28348410

[B28] Ma J. Bao Y. Sun M. Dong W. Pan Z. Zhang W. (2014). Two functional type VI secretion systems in avian pathogenic *Escherichia coli* are involved in different pathogenic pathways. *Infect. Immun.* 82 3867–3879. 10.1128/IAI.01769-1714 24980972 PMC4187841

[B29] Mendez V. Agullo L. Gonzalez M. Seeger M. (2011). The homogentisate and homoprotocatechuate central pathways are involved in 3- and 4-hydroxyphenylacetate degradation by *Burkholderia xenovorans* LB400. *PLoS One* 6:e17583 10.1371/journal.pone.0017583 PMC3053370 21423751

[B30] Miller J. H. (1992). *A Short Course in Bacterial Genetics - A Laboratory Manual and Handbook for Escherichia coli and Related Bacteria.* Cold Spring Harbor, NY: Cold Spring Harbor Laboratory Press.

[B31] Navarro-Garcia F. Ruiz-Perez F. Cataldi A. Larzabal M. (2019). Type VI secretion system in pathogenic *Escherichia coli*: structure, role in virulence, and acquisition. *Front. Microbiol.* 10:1965 10.3389/fmicb.2019.01965 PMC6730261 31543869

[B32] Perez-Rueda E. Collado-Vides J. (2001). Common history at the origin of the position-function correlation in transcriptional regulators in archaea and bacteria. *J. Mol. Evol.* 53 172–179. 10.1007/s002390010207 11523004

[B33] Perez-Rueda E. Collado-Vides J. Segovia L. (2004). Phylogenetic distribution of DNA-binding transcription factors in bacteria and archaea. *Comput. Biol. Chem.* 28 341–350. 10.1016/j.compbiolchem.2004.09.004 15556475

[B34] Prieto M. A. Diaz E. Garcia J. L. (1996). Molecular characterization of the 4-hydroxyphenylacetate catabolic pathway of *Escherichia coli* W: engineering a mobile aromatic degradative cluster. *J. Bacteriol.* 178 111–120. 10.1128/jb.178.1.111-120.1996 8550403 PMC177627

[B35] Prieto M. A. Garcia J. L. (1994). Molecular characterization of 4-hydroxyphenylacetate 3-hydroxylase of *Escherichia coli*. A two-protein component enzyme. *J. Biol. Chem.* 269 22823–22829. 8077235

[B36] Prieto M. A. Garcia J. L. (1997). Identification of a novel positive regulator of the 4-hydroxyphenylacetate catabolic pathway of *Escherichia coli*. *Biochem. Biophys. Res. Commun.* 232 759–765. 10.1006/bbrc.1997.6368 9126350

[B37] Providenti M. A. Wyndham R. C. (2001). Identification and functional characterization of CbaR, a MarR-like modulator of the cbaABC-encoded chlorobenzoate catabolism pathway. *Appl. Environ. Microbiol.* 67 3530–3541. 10.1128/AEM.67.8.3530-3541.2001 11472929 PMC93053

[B38] Roper D. I. Fawcett T. Cooper R. A. (1993). The *Escherichia coli* C homoprotocatechuate degradative operon: hpc gene order, direction of transcription and control of expression. *Mol. Gen. Genet.* 237 241–250. 10.1007/bf00282806 8384293

[B39] Russell A. B. Peterson S. B. Mougous J. D. (2014). Type VI secretion system effectors: poisons with a purpose. *Nat. Rev. Microbiol.* 12 137–148. 10.1038/nrmicro3185 24384601 PMC4256078

[B40] Shen X. Banga S. Liu Y. Xu L. Gao P. Shamovsky I. (2009). Targeting eEF1A by a *Legionella pneumophila* effector leads to inhibition of protein synthesis and induction of host stress response. *Cell Microbiol.* 11 911–926. 10.1111/j.1462-5822.2009.01301.x 19386084 PMC2967282

[B41] Shen X. H. Jiang C. Y. Huang Y. Liu Z. P. Liu S. J. (2005). Functional identification of novel genes involved in the glutathione-independent gentisate pathway in *Corynebacterium glutamicum*. *Appl. Environ. Microbiol.* 71 3442–3452. 10.1128/AEM.71.7.3442-3452.2005 16000747 PMC1169049

[B42] Si M. Wang T. Pan J. Lin J. Chen C. Wei Y. (2017a). Graded response of the multifunctional 2-cysteine peroxiredoxin, CgPrx, to increasing levels of hydrogen peroxide in *Corynebacterium glutamicum*. *Antioxid. Redox. Signal.* 26 1–14. 10.1089/ars.2016.6650 27324811

[B43] Si M. Wang Y. Zhang B. Zhao C. Kang Y. Bai H. (2017b). The type VI secretion system engages a redox-regulated dual-functional heme transporter for zinc acquisition. *Cell Rep.* 20 949–959. 10.1016/j.celrep.2017.06.081 28746878

[B44] Si M. Zhao C. Burkinshaw B. Zhang B. Wei D. Wang Y. (2017c). Manganese scavenging and oxidative stress response mediated by type VI secretion system in *Burkholderia thailandensis*. *Proc. Natl. Acad. Sci. U.S.A.* 114 E2233–E2242. 10.1073/pnas.1614902114 28242693 PMC5358365

[B45] Si M. Zhang L. Chaudhry M. T. Ding W. Xu Y. Chen C. (2015). *Corynebacterium glutamicum* methionine sulfoxide reductase A uses both mycoredoxin and thioredoxin for regeneration and oxidative stress resistance. *Appl. Environ. Microbiol.* 81 2781–2796. 10.1128/AEM.04221-4214 25681179 PMC4375309

[B46] Slauch J. M. (2011). How does the oxidative burst of macrophages kill bacteria? Still an open question. *Mol. Microbiol.* 80 580–583. 10.1111/j.1365-2958.2011.07612.x 21375590 PMC3109634

[B47] Smego R. A. Frean J. Koornhof H. J. (1999). Yersiniosis I: microbiological and clinicoepidemiological aspects of plague and non-plague Yersinia infections. *Eur. J. Clin. Microbiol. Infect. Dis.* 18 1–15. 10.1007/s100960050219 10192708

[B48] Song Y. Xiao X. Li C. Wang T. Zhao R. Zhang W. (2015). The dual transcriptional regulator RovM regulates the expression of AR3- and T6SS4-dependent acid survival systems in response to nutritional status in *Yersinia pseudotuberculosis*. *Environ. Microbiol.* 17 4631–4645. 10.1111/1462-2920.12996 26234561

[B49] Staerck C. Gastebois A. Vandeputte P. Calenda A. Larcher G. Gillmann L. (2017). Microbial antioxidant defense enzymes. *Microb. Pathog.* 110 56–65. 10.1016/j.micpath.2017.06.015 28629723

[B50] Tan Y. Liu W. Zhang Q. Cao S. Zhao H. Wang T. (2017). *Yersinia pestis* YopK inhibits bacterial adhesion to host cells by binding to the extracellular matrix adaptor protein matrilin-2. *Infect. Immun.* 85:e01069-16 10.1128/IAI.01069-1016 PMC5520434 28533472

[B51] Wan B. Zhang Q. Ni J. Li S. Wen D. Li J. (2017). Type VI secretion system contributes to enterohemorrhagic *Escherichia coli* virulence by secreting catalase against host reactive oxygen species (ROS). *PLoS Pathog* 13:e1006246. 10.1371/journal.ppat.1006246 28288207 PMC5363993

[B52] Wang T. Chen K. Gao F. Kang Y. Chaudhry M. T. Wang Z. (2017). ZntR positively regulates T6SS4 expression in *Yersinia pseudotuberculosis*. *J. Microbiol.* 55 448–456. 10.1007/s12275-017-6540-6542 28281200

[B53] Wang T. Si M. Song Y. Zhu W. Gao F. Wang Y. (2015). Type VI secretion system transports Zn2+ to combat multiple stresses and host immunity. *PLoS Pathog.* 11:e1005020 10.1371/journal.ppat.1005020 PMC4489752 26134274

[B54] Wang Y. Cen X. F. Zhao G. P. Wang J. (2012). Characterization of a new GlnR binding box in the promoter of amtB in *Streptomyces coelicolor* inferred a PhoP/GlnR competitive binding mechanism for transcriptional regulation of amtB. *J. Bacteriol.* 194 5237–5244. 10.1128/JB.00989-912 22821977 PMC3457235

[B55] Weber B. Hasic M. Chen C. Wai S. N. Milton D. L. (2009). Type VI secretion modulates quorum sensing and stress response in *Vibrio anguillarum*. *Environ. Microbiol.* 11 3018–3028. 10.1111/j.1462-2920.2009.02005.x 19624706

[B56] Wei K. Tang D. J. He Y. Q. Feng J. X. Jiang B. L. Lu G. T. (2007). hpaR, a putative marR family transcriptional regulator, is positively controlled by HrpG and HrpX and involved in the pathogenesis, hypersensitive response, and extracellular protease production of *Xanthomonas campestris* pathovar campestris. *J. Bacteriol.* 189 2055–2062. 10.1128/JB.01331-1336 17158655 PMC1855773

[B57] Xu L. Shen X. Bryan A. Banga S. Swanson M. S. Luo Z. Q. (2010). Inhibition of host vacuolar H+-ATPase activity by a *Legionella pneumophila* effector. *PLoS Pathog.* 6:e1000822 10.1371/journal.ppat.1000822 PMC2841630 20333253

[B58] Xu S. Peng Z. Cui B. Wang T. Song Y. Zhang L. (2014). FliS modulates FlgM activity by acting as a non-canonical chaperone to control late flagellar gene expression, motility and biofilm formation in *Yersinia pseudotuberculosis*. *Environ. Microbiol.* 16 1090–1104. 10.1111/1462-2920.12222 23957589

[B59] Yang X. Pan J. Wang Y. Shen X. (2018). Type VI secretion systems present new insights on pathogenic yersinia. *Front. Cell Infect. Microbiol.* 8:260 10.3389/fcimb.2018.00260 PMC6079546 30109217

[B60] Zhang W. Wang Y. Song Y. Wang T. Xu S. Peng Z. (2013). A type VI secretion system regulated by OmpR in *Yersinia pseudotuberculosis* functions to maintain intracellular pH homeostasis. *Environ. Microbiol.* 15 557–569. 10.1111/1462-2920.12005 23094603

[B61] Zhao R. Song Y. Dai Q. Kang Y. Pan J. Zhu L. (2017). A starvation-induced regulator, RovM, acts as a switch for planktonic/biofilm state transition in *Yersinia pseudotuberculosis*. *Sci. Rep.* 7:639 10.1038/s41598-017-00534-539 PMC5428675 28377623

