# Supplementary material for: HpaR, the Repressor of Aromatic Compound Metabolism, Positively Regulates the Expression of T6SS4 to Resist Oxidative Stress in Yersinia pseudotuberculosis
Source: Front Microbiol. 2020 Apr 17;11:705. doi: 10.3389/fmicb.2020.00705 (PMC7180172; doi:10.3389/fmicb.2020.00705)
Supplement: FIGURE S1 — Protein sequence alignment of HpaR homologs by CLUSTAL W. The sequences used in alignment have been deposited in the GenBank database [Accession No. EcC Escherichia coli C (S56952.1), EcW Escherichia coli W (Z37980.2), Yptb Yersinia pseudotuberculosis YPIII (ACA68739.1), PpU Pseudomonas putida U (FJ904934.1), BxLB400 Burkholderia xenovorans LB400 (ABE33958.1)]. The result was exported by ESPript (http://espript.ibcp.fr/ESPript/cgi-bin/ESPript.cgi). [file Data_Sheet_1.PDF]

Supplementary Table S1. The strains and plasmids used in this study.

Strain or plasmid	Relevant characteristics	Reference
E. coli		
S17-1λ *pir*	λ-pir lysogen of S17-1, *thi pro hsdR hsdM*$^+$ *recA* RP4 2-Tc::Mu-Km::Tn7	(Simon, et al. 1983)
BL21(DE3)	Host for expression vector pET28a	Novagen
Y. pseudotuberculosis		
Yptb YPIII	Wild-type *Y. pseudotuberculosis*, Nal^r	(Rosqvist, et al. 1988)
*Yptb*Δ*hpaR*	*hpaR* gene deleted in *Yptb*, Nal^r	This study
*Yptb*Δ*hpaR*Δ*clpv4*	*hpaR* and *clpV4* gene deleted in *Yptb*, Nal^r	This study
Yptb WT(Vector)	*Yptb* Wild-type containing pKT100, Nal^r, Km^r	(Zhang, et al. 2013)
*Yptb*Δ*hpaR*(Vector)	*Yptb*Δ*hpaR* containing pKT100, Nal^r, Km^r	This study
*Yptb*Δ*hpaR*(*hpaR*)	*Yptb*Δ*clpV4* containing pKT100-*hpaR*, Nal^r, Km^r	This study
*Yptb*Δ*hpaR*Δ*clpv4*(Vector)	*Yptb*Δ*hpaR*Δ*clpv4* containing pKT100, Nal^r, Km^r	This study
*Yptb*Δ*hpaR*Δ*clpv4*(*hpaR*)	*Yptb*Δ*hpaR*Δ*clpv4* containing pKT100-*hpaR*, Nal^r, Km^r	This study
**Plasmid**		
pKT100	Cloning vector, p15A replicon, Km^r	(Hu, et al. 2009)
pKT100-*hpaR*	*hpaR* under the control of chloramphenicol resistance gene promoter in plasmid pKT100	This study
pET28a	Expression vector with N-terminal hexahistidine affinity tag, Km^r	Novagen
pET28a-*hpaR*	pET28a carrying *hpaR* coding region, Km^r	This study
pME6032	Shuttle vector, Tc^r	(Zhao and

		Shao 2015)
pME6032-*yezP-vsvg*	pME6032 carrying *yezP-vsvg* coding region, Tc^r	(Wang, et al. 2015)
pDM4	Suicide vector, *mob*RK2, *ori*R6K, *pir*, *sacB*, Cm^r	(Milton, et al. 1996)
pDM4-Δ*hpaR*	Construct used for in-frame deletion of *hpaR*, Cm^r	This study
pDM4-*hpaG1p::lacZ*	For *hpaG1* promoter fusion to *Yptb*, Cm^r	This study
pDM4-T6SS4*p*::*lacZ*	For T6SS4 promoter fusion to *Yptb*, Cm^r	This study
pDM4-T6SS4*p*M*::lacZ*	T6SS4*p*M*::lacZ*, HpaR binding site mutated, Cm^r	This study

*Nal^r, Cm^r, Km^r, Tc^r and Amp^r represent resistance to naladixic acid, chloramphenicol, kanamycin, tetracycline and ampicillin at 15, 30, 50, 10 and 100 μg/mL, respectively.

Supplementary Table S2. Primers used in this study.

Primers	5'-3' sequence	Function
hpaR-UF-*Xba*I	CTAGTCTAGAATGGCCTACTTCCTTGCTGG	To generate pDM4-Δ*hpaR*
hpaR-UR	**ACTCTTCATCCAGTGATTCA**TGCATAAGTA	
hpaR-DF	**TGAATCACTGGATGAAGAGT**GATGGGTTTA	
hpaR-DR-*Spe*I	CTAGACTAGTTTCTCAATCTTGATTGAGTT	
hpaR-F-*Bam*HI	CGCGGATCCATGCATGAATCACTGACCAT	To generate pKT100-*hpaR*, pET28a-*hpaR*
hpaR-R-*Sal*I	ACGCGTCGACTCACTCTTCATCTTCTAACA	
HpaG1-QF	ATGACTATGCCGTTCGTGAC	qRT-PCR
HpaG1-QR	ATTGGGAATATCCGCTTTAT	
hpaE-QF	TCCCTCACTGGGCTAACCTG	
hpaE-QR	TATCCTGCGTTTCCATCTGC	
hpaB-QF	GTGTCCAAGCGGATTTAGGT	
hpaB-QR	CTGCGAGATCGGGTAGATAA	
vgrG4-QF	AGGGAATCCATCCTACCA	
vgrG4-QR	AATTTGTCTTGCCGTTGC	
hcp4-QF	GTAACTGTCTGGTGTCCTCC	
hcp4-QR	CCATCAGGTTGCTGCTCT	
clpV4-QF	GGCGTCACCTTCTCCTATC	
clpV4-QR	TGAACCTCGCTGGTCTGT	
YPK_0997-QF	CACTGGAGGAAGGTGATTGG	
YPK_0997-QR	CGGGATTTATCATTGGCAGA	
YPK_2453-QF	GTGTCCAAGCGGATTTAGGT	
YPK_2453-QR	CTGCGAGATCGGGTAGATAA	
YPK_2458-QF	TTCAGCGAGTTCTGCCAAAT	
YPK_2458-QR	TTATCCCATCCCAGCATCCC	

YPK_3395-QF	CACGTCATGCCACGATTTGG	
YPK_3395-QR	TTCTGCCTCGGTCATCAAGC	
YPK_3402-QF	CGAACCGCAGGATAGTGAAA	
YPK_3402-QR	TCCATGATCTCGACCGCACA	
YPK_4175-QF	AGGCACGGTGGTATTAGAGG	
YPK_4175-QR	GGAGCGATGTCCCATTGTTT	
YPK_2561-QF	CATACAGCCAATCCCACAGT	
YPK_2561-QR	GGGCAGGCTAGGGTTAAAGG	
YPK_3558-QF	GTATTGGGACGGTAGGAAGC	
YPK_3558-QR	TTGAGCGCCGGTATAACTGA	
YPK_3559-QF	CGGCATGAGTATGTGGAGCT	
YPK_3559-QR	GCGTGGAGATAACCGTCTGG	
YPK_3624-QF	TCTACACGCCAGACCCACAA	
YPK_3624-QR	GCACCAAACTCTGCCGCTAC	
YPK_3776-QF	TTTAACTATGGGCTGGACTA	
YPK_3776-QR	ATCAGGTAAGGCTGGAACAA	
16S RNA-F	CTAGCGATTCCGACTTCAT	
16S RNA-R	CCCTTATCCTTTGTTGCC	
T6SS4*p*-FP-F	AGAAGGCGTTGATGTTTGAC	HpaR Footprinting
T6SS4*p*-FP-R	AACGCCGAATAATGCTTGAG	
Bio-T6SS4*p*$_{\mathrm{HpaR}}$-F	GTGGATTTCGCCTCAGGCAT	EMSA
Bio-T6SS4*p*$_{\mathrm{HpaR}}$-R	CATCCTGATTTACATACCTG	
T6SS4*p*$_{\mathrm{HpaR}}$-F	GTGGATTTCGCCTCAGGCAT	
T6SS4*p*$_{\mathrm{HpaR}}$-R	CATCCTGATTTACATACCTG	
T6SS4*p*$_{\mathrm{HpaR}}$-F-*Sal*I	ACGC<u>GTCGAC</u>GTGGATTTCGCCTCAGGCAT	To generate pDM4-T6SS4*p*$_{\mathrm{HpaR}}$*::lacZ*
T6SS4*p*M$_{\mathrm{HpaR}}$-F	**ATTTGTTAGATTCCGAACCGTC**ATCGTGCTAATGGTTATG	

T6SS4p_{HpaR}M-R	**GACGGTTCGGAATCTAACAAAT**AAGAGGACATAGATATGAGAAAGA	
T6SS4p_{HpaR}-R-*Xba*I	CTAGTCTAGACATCCTGATTTACATACCTG	
hpaG1p-FP-F	CTGCAATAATGCGATGGTCA	HpaR Footprinting
hpaG1p-FP-R	TTCAATGCAACGGCAAATAC	
hpaG1p-F	AAGTAACAACGACTCGACGT	EMSA
hpaG1p-R	GCAATGTTCCTTTTGGCTTA	
hpaG1p-F-*Sal*I	ACGCGTCGACCTGCAATAATGCGATGGTCA	To generate pDM4-*hpaG1p::lacZ*
hpaG1p-R-*Xba*I	CTAGTCTAGATTCAATGCAACGGCAAATAC	

Underlined sites indicate restriction enzyme cutting sites added for cloning. Letters in boldface denote the annealing regions for overlap PCR.

Supplementary Table S3. Genes differentially transcribed in Δ*hpaR* mutant compared to the *Y. pseudotuberculosis* wild-type detected by RNA-seq.

CDS	Gene	Predicted function	[a]Fold change
YPK_0033		hypothetical protein	2.64
YPK_0153		hypothetical protein	3.18
YPK_0442		cold shock protein	2.00
YPK_0774		hypothetical protein	2.11
YPK_0794		lipoprotein	1.65
YPK_0795		insertion element IS1 protein	3.11
YPK_0796		hypothetical protein	4.36
YPK_0833	*pilT*	twitching motility protein	1.65
YPK_0847		penicillin-binding protein 1C	7.63
YPK_0954		hypothetical protein	3.23
**YPK_0997**		**chondroitin-sulfate-ABC endolyase/exolyase**	**1.66**
YPK_0998		subtilase-type serine protease	2.09
YPK_1008		general secretion pathway protein H	3.86
YPK_1037		prepilin peptidase dependent protein A	2.74
YPK_1121		hypothetical protein	1.82
YPK_1160		hypothetical protein	1.87
YPK_1238		hypothetical protein	2.05
YPK_1244		hypothetical protein	7.18
YPK_1254		hypothetical protein	1.56
YPK_1317		protein of unknown function DUF943	1.61
YPK_1359		hypothetical protein	1.53
YPK_1395		hypothetical protein	4.17
YPK_1601		protein of unknown function DUF218	1.59
YPK_2452	*hpaC*	4-hydroxyphenylacetate-3-monooxygenase	2.31
**YPK_2453**	***hpaB***	**4-hydroxyphenylacetate-3-monooxygenase**	**3.24**
YPK_2454	*hpaX*	4-hydroxyphenylacetate permease	3.37
YPK_2455	*hpaI*	2,4-dihydroxyhept-2-ene-1,7-dioic acid aldolase	3.83
YPK_2456	*hpaH*	2-oxo-hept-3-ene-1,7-dioate hydratase	3.91
YPK_2457	*hpaF*	5-carboxymethyl-2-hydroxymuconate isomerase	4.25
**YPK_2458**	***hpaD***	**3,4-dihydroxyphenylacetate 2,3-dioxygenase**	**4.42**
YPK_2459	*hpaE*	5-carboxymethyl-2-hydroxymuconic-	4.45
YPK_2460	*hpaG2*	2-hydroxyhepta-2,4-diene-1,7-dioate isomerase	4.56
YPK_2461	*hpaG1*	2-hydroxyhepta-2,4-diene-1,7-dioate isomerase	4.50
YPK_2494		hypothetical protein	1.53
YPK_2604		hypothetical protein	2.00
YPK_2893		hypothetical protein	1.58
YPK_2895		8-oxo-dGTP diphosphatase	7.34
YPK_3508		hypothetical protein	1.54
YPK_3098	*yscL*	type III secretion apparatus protein	2.71
YPK_3229	*comE*	competence protein	2.42
YPK_3294		transcriptional regulator, LysR family	2.04
YPK_3359	*dcuB*	anaerobic C4-dicarboxylate transporter	2.57
**YPK_3395**		**L-ribulose-5-phosphate 4-epimerase**	**1.52**

YPK_3397		DeoR family transcriptional regulator	2.56
YPK_3398		simple sugar transport system substrate-binding	1.80
YPK_3399		simple sugar transport system ATP-binding	2.59
YPK_3400		simple sugar transport system permease protein	2.02
YPK_3401		simple sugar transport system permease protein	2.28
**YPK_3402**		**hexulose-6-phosphate isomerase**	**2.13**
YPK_3403		L-xylulokinase	2.53
YPK_3414		hypothetical protein	1.95
YPK_3653		AI-2 transport system substrate-binding protein	1.88
YPK_3654		putative autoinducer-2 (AI-2) aldolase	1.87
YPK_3655		autoinducer 2-degrading protein	2.31
YPK_3748		hypothetical protein	6.19
YPK_3915		type III secretion system protein	2.91
**YPK_4175**		**DNA ligase (NAD+)**	**1.60**
YPK_0238	*tsgA*	MFS transporter	-3.88
YPK_0382		maltose operon periplasmic protein	-2.31
YPK_0678		tight adherence protein E	-3.54
YPK_0680		tight adherence protein C	-2.66
YPK_0681		tight adherence protein B	-4.20
YPK_0773		hypothetical protein	-1.56
YPK_0789		protocatechuate 3,4-dioxygenase	-1.61
YPK_0886		hypothetical protein	-6.07
YPK_1011		general secretion pathway protein K	-4.03
YPK_1013		hypothetical protein	-3.58
YPK_1269		3-phenylpropionic acid transporter	-2.03
YPK_1438		nucleoside transport protein	-3.33
YPK_1584		hypothetical protein	-5.59
YPK_1761	*hsf*	adhesin	-2.14
YPK_2286		putative transposase	-6.24
YPK_2297		GPW/gp25 family protein	-1.87
YPK_2316		hypothetical protein	-5.98
YPK_2341		phage baseplate assembly protein V	-2.83
YPK_2462	*hpaR*	MarR family transcriptional regulator	-11.55
**YPK_2561**		**cytidine deaminase**	**-5.25**
YPK_2588		hypothetical protein	-4.52
YPK_2720		iron complex transport system substrate-binding	-1.93
YPK_3534			-4.74
YPK_3550	*impL*	type VI secretion system protein	-1.52
YPK_3551	*impK*	type VI secretion system protein	-1.53
YPK_3552	*impJ*	type VI secretion system protein	-1.63
YPK_3555		hypothetical protein	-1.77
YPK_3556		adenylate cyclase	-1.74
YPK_3557		adenylate cyclase	-1.77
**YPK_3558**	***vgrG***	**type VI secretion system secreted protein**	**-1.78**
**YPK_3559**	***vasG***	**type VI secretion system protein**	**-1.70**
YPK_3560	*impH*	type VI secretion system protein	-1.52
YPK_3561	*impG*	type VI secretion system protein	-1.79
YPK_3564	*impC*	type VI secretion system protein	-1.77
YPK_3565	*impB*	type VI secretion system protein	-1.69

YPK_3566	*impA*	type VI secretion system protein	-1.90
YPK_3623		membrane protein	-1.76
**YPK_3624**		**purine-nucleoside phosphorylase**	**-3.62**
YPK_3625		phosphopentomutase	-4.05
YPK_3626		thymidine phosphorylase	-4.38
YPK_3627		deoxyribose-phosphate aldolase	-4.05
YPK_3628		concentrative nucleoside transporter, CNT	-2.70
YPK_3670		hypothetical protein	-2.14
YPK_3724		ATP-dependent RNA helicase	-1.60
**YPK_3776**		**2',3'-cyclic-nucleotide 2'-phosphodiesterase**	**-3.63**
YPK_3844		phosphoribosylaminoimidazol (AIR) synthetase	-7.68
YPK_3912		type III secretion system protein	-5.56
YPK_3949		protein-tyrosine phosphatase	-2.77
YPK_3950		uridine phosphorylase	-4.87
YPK_4107		hypothetical protein	-2.32

RNA-seq-based transcriptomics analysis was performed using total RNAs isolated from *Y. pseudotuberculosis* YPIII Δ*hpaR* mutant compared to the *Y. pseudotuberculosis* YPIII wild-type. The genes that are at least 1.5-fold changed in biological replicates were considered as significant. T6SS4 genes and the *hpa-meta* gene were highlighted in yellow. qRT-PCR verified genes were shown in boldface. [a] Fold change was defined by $2^{(\text{the gene expression ratio of } \Delta hpaR \text{ mutant to the } Y.\ pseudotuberculosis \text{ YPIII wild-type})}$.

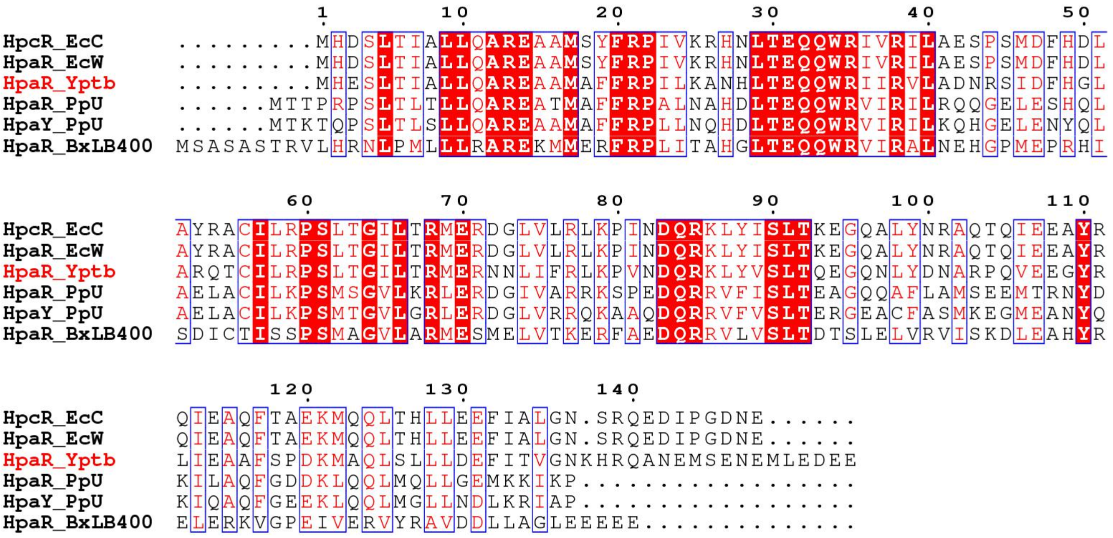

Fig. S1. Protein sequence alignment of HpaR homologues by CLUSTAL W.

The sequences used in alignment have been deposited in the GenBank database (Accession No. EcC *Escherichia coli* C (S56952.1), EcW *Escherichia coli* W (Z37980.2), Yptb *Yersinia pseudotuberculosis* YPIII (ACA68739.1), PpU *Pseudomonas putida* U (FJ904934.1), BxLB400 *Burkholderia xenovorans* LB400 (ABE33958.1)). The result was exported by ESPript (http://espript.ibcp.fr/ESPript/cgi-bin/ESPript.cgi).

References:

1. Simon, R., Priefer, U. and Pühler, A.J.B.t. 1983 A broad host range mobilization system for in vivo genetic engineering: transposon mutagenesis in gram negative bacteria. **1** (9), 784.
2. Rosqvist, R., Skurnik, M. and Wolf-Watz, H. 1988 Increased virulence of Yersinia pseudotuberculosis by two independent mutations. *Nature*, **334** (6182), 522-524.
3. Zhang, W., Wang, Y., Song, Y., Wang, T., Xu, S., Peng, Z. *et al.* 2013 A type VI secretion system regulated by OmpR in Yersinia pseudotuberculosis functions to maintain intracellular pH homeostasis. *Environmental microbiology*, **15** (2), 557-569.
4. Hu, Y., Lu, P., Wang, Y., Ding, L., Atkinson, S. and Chen, S. 2009 OmpR positively regulates urease expression to enhance acid survival of Yersinia pseudotuberculosis. *Microbiology*, **155** (Pt 8), 2522-2531.
5. Zhao, Y. and Shao, F. 2015 The NAIP-NLRC4 inflammasome in innate immune detection of bacterial flagellin and type III secretion apparatus.

Immunol Rev, **265** (1), 85-102.

6. Wang, T., Si, M., Song, Y., Zhu, W., Gao, F., Wang, Y. *et al.* 2015 Type VI Secretion System Transports Zn2+ to Combat Multiple Stresses and Host Immunity. *PLoS pathogens*, **11** (7), e1005020.
7. Milton, D.L., O'Toole, R., Horstedt, P. and Wolf-Watz, H. 1996 Flagellin A is essential for the virulence of Vibrio anguillarum. *Journal of bacteriology*, **178** (5), 1310-1319.
