# Supplementary figures and images for: HpaR, the Repressor of Aromatic Compound Metabolism, Positively Regulates the Expression of T6SS4 to Resist Oxidative Stress in Yersinia pseudotuberculosis
Source: Front Microbiol. 2020 Apr 17;11:705. doi: 10.3389/fmicb.2020.00705 (PMC7180172; doi:10.3389/fmicb.2020.00705)

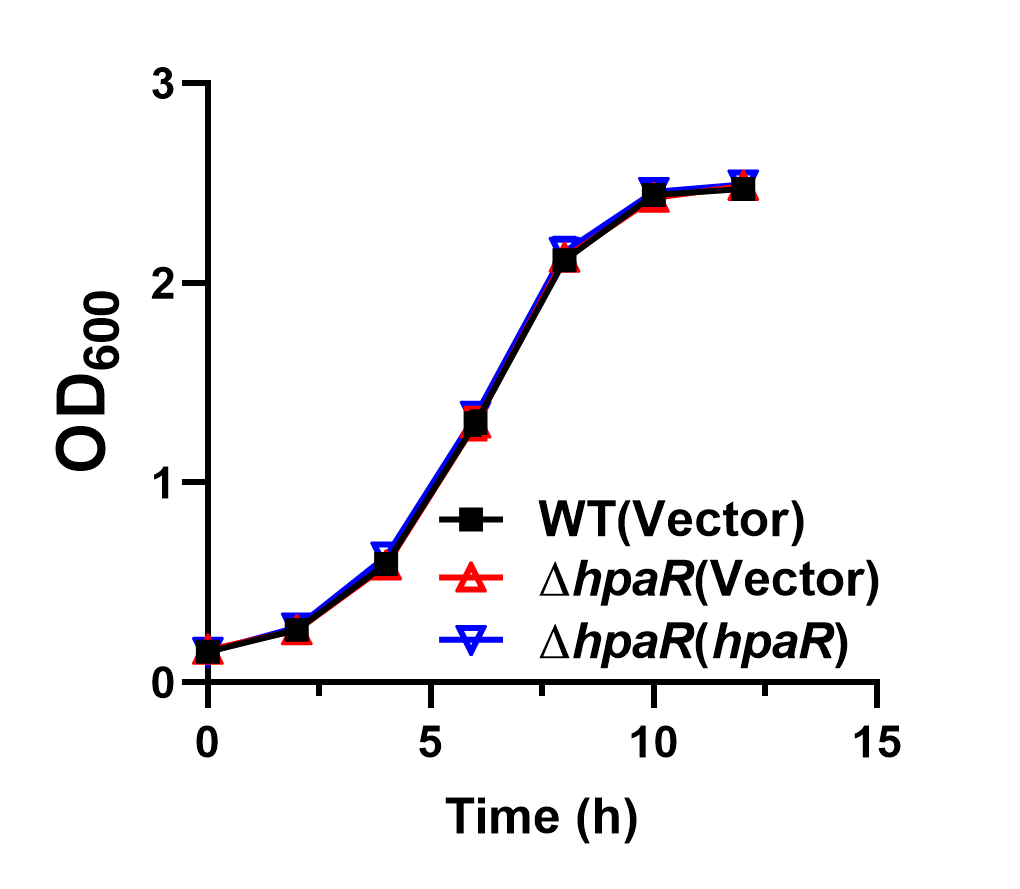

Supplement: FIGURE S2 — Growth curves of the wild-type (WT), ΔhpaR mutant, and the complemented strain ΔhpaR (hpaR) under normal condition. The growth of the indicated strains in YLB medium was monitored by measuring OD600 at indicated time points under 26°C. [file Data_Sheet_2.zip › Figure S2.TIF]
